# Pre-treatment endocrine–nutritional signatures predict clinical benefit from PD-1/PD-L1 blockade in hematologic malignancies

**DOI:** 10.3389/fnut.2025.1753660

**Published:** 2026-02-05

**Authors:** Ningjing Huang, Yu Guan

**Affiliations:** 1 Department of Neurology, Shanghai Municipal Hospital of Traditional Chinese Medicine, Shanghai University of Traditional Chinese Medicine, Shanghai, China; 2 Diagnostic Laboratory for Hematology, Shanghai Municipal Hospital of Traditional Chinese Medicine, Shanghai University of Traditional Chinese Medicine, Shanghai, China

**Keywords:** endocrine signatures, hematologic malignancies, immune checkpoint inhibitors, immunotherapy outcomes, nutritional biomarkers, PD-1/PD-L1 blockade

## Abstract

Hematologic malignancies pose significant global health burdens, with programmed cell death protein-1 (PD-1)/programmed cell death ligand 1 (PD-L1) inhibitors revolutionizing treatment in subtypes like classical Hodgkin lymphoma (cHL) and primary mediastinal large B-cell lymphoma (PMBCL), achieving high objective response rates (ORR). However, efficacy varies widely, with limited success in multiple myeloma (< 10% ORR) and leukemias, underscoring the need for better predictors beyond tumor-intrinsic biomarkers. This review highlights pre-treatment endocrine–nutritional signatures as key host factors influencing immunotherapy outcomes. Dysregulated hormones (cortisol, thyroid, sex steroids, insulin/insulin-like growth factor-1, adipokines) and nutritional status (vitamin D, zinc, protein-energy malnutrition, iron metabolism) modulate T-cell exhaustion, myeloid suppression, and tumor microenvironment dynamics, often leading to resistance. Evidence from cohorts shows hypercortisolism, hypothyroidism, insulin resistance, vitamin D deficiency, and hypoalbuminemia correlate with inferior ORR, progression-free survival, and overall survival, while thyroid immune-related adverse events and moderate obesity predict benefit. In hematologic contexts, marrow infiltration exacerbates these imbalances, explaining heterogeneous responses. Integrated signatures (e.g., Glasgow Prognostic Score, Prognostic Nutritional Index) offer superior prognostic value, enabling targeted interventions like vitamin D supplementation, metformin, or nutritional support to enhance immune checkpoint inhibitor efficacy. Mechanistic insights reveal convergence on mTOR/IFN-γ pathways and microbiome modulation. Translating these to clinical panels could personalize immunotherapy, addressing gaps in hematologic malignancies literature and improving outcomes in relapsed/refractory settings.

## 1 Introduction

Hematologic malignancies encompassing lymphomas, leukemias, multiple myeloma (MM), and related disorders represent a major global health challenge, with approximately 1.3 million new cases and 700,000 deaths annually according to GLOBOCAN 2022 estimates, projected to rise substantially by 2050 due to population aging and environmental exposures (1, 2). These cancers disproportionately affect younger populations in low- and middle-income countries and carry high morbidity from disease- and treatment-related immunosuppression (3). Traditional therapies (chemotherapy, targeted agents, hematopoietic stem cell transplantation) have improved survival but plateaued in relapsed/refractory (r/r) settings, prompting a paradigm shift toward immunotherapy, particularly immune checkpoint inhibitors (ICIs) targeting the programmed cell death protein-1 (PD-1)/programmed cell death ligand 1 (PD-L1) axis (4). Since the landmark approvals of nivolumab and pembrolizumab for r/r classical Hodgkin lymphoma (cHL) in 2016–2017, PD-1 blockade has transformed outcomes in select subtypes, achieving objective response rates (ORR) of 70–87% and durable remissions in cHL and primary mediastinal large B-cell lymphoma (PMBCL), with expanding roles in combinations for non-Hodgkin lymphomas and post-transplant relapse (5, 6).

Despite these successes, responses to PD-1/PD-L1 inhibitors remain strikingly heterogeneous across hematologic malignancies: exquisite sensitivity in 9p24.1-altered cHL/PMBCL contrasts with modest activity in T-cell lymphomas (ORR 20–50%), negligible monotherapy efficacy in MM (< 10%), and limited benefit in leukemias outside niche indications (7, 8). Even within responsive diseases, 20–40% of patients exhibit primary resistance or early relapse, highlighting the inadequacy of tumor-centric biomarkers alone to explain variability (9).

This inconsistency has fueled recognition that tumor-intrinsic features (PD-L1 expression, tumor mutational burden, genetic alterations) are insufficient predictors in many hematological contexts, directing attention to host systemic factors that establish a baseline “whole-body immunologic tone,” the integrated metabolic, endocrine, and inflammatory milieu shaping T-cell priming, trafficking, and persistence (10).

Hormones (cortisol, thyroid hormones, sex steroids, insulin/Insulin-like growth factor-1 (IGF-1), adipokines), micronutrients (vitamin D, zinc, selenium), and metabolic state (obesity, sarcopenia, protein-energy status) directly modulate T-cell metabolism, exhaustion, and cytokine networks, influencing whether PD-1/PD-L1 blockade can restore effective antitumor immunity (11, 12). Pre-treatment dysregulation, highly prevalent in hematologic patients due to disease cachexia, marrow infiltration, and prior therapies, correlates with inferior ICI outcomes across cancers, identifying a modifiable determinant of response (13). Assessing these signatures before therapy offers prognostic value and therapeutic opportunity through targeted interventions.

This review synthesizes emerging evidence linking pre-treatment endocrine–nutritional profiles to PD-1/PD-L1 blockade efficacy in hematologic malignancies, emphasizing mechanistic insights, clinical correlations, and translational potential beyond solid tumor-dominated literature.

## 2 PD-1/PD-L1 blockade in hematologic malignancies: a clinical overview

PD-1/PD-L1 inhibitors have revolutionized relapsed/refractory cHL and PMBCL with high, durable response rates, leading to regulatory approvals for nivolumab and pembrolizumab. Efficacy remains heterogeneous elsewhere: modest in select non-Hodgkin lymphomas [particularly T-cell or Epstein-Barr virus (EBV)-associated], negligible in MM as monotherapy, and emerging but limited in leukemias (mainly post-transplant relapse). Predictive biomarkers such as PD-L1 expression (driven by 9p24.1 alterations in cHL/PMBCL), tumor mutational burden (TMB), and genetic features perform well in cHL but have major limitations in other hematologic settings due to low neoantigen burden, immunosuppressive microenvironments, assay variability, and confounding inflammation.

### 2.1 Mechanistic basis of immune checkpoint inhibition

The PD-1 receptor on T cells interacts with its ligands PD-L1 (CD274) and PD-L2 (CD273) on tumor cells or antigen-presenting cells, delivering inhibitory signals that induce T-cell exhaustion, energy, and apoptosis mechanisms that tumors exploit for immune evasion (14). PD-1/PD-L1 blockade with monoclonal antibodies (e.g., nivolumab, pembrolizumab targeting PD-1; atezolizumab, durvalumab targeting PD-L1) disrupts this axis, restoring effector T-cell function, cytokine production (IFN-γ), and cytolytic activity (15).

In hematologic malignancies, Reed-Sternberg (RS) cells in cHL and malignant B cells in PMBCL frequently harbor copy number alterations at chromosome 9p24.1 encompassing PD-L1/PD-L2 and JAK2, leading to JAK/STAT-mediated overexpression of PD-L1/PD-L2 and profound dependence on the pathway for survival (9). This genetic addiction renders these tumors exquisitely sensitive to PD-1 blockade, resulting in one of the highest response rates observed across oncology (ORR 69–87%) (5, 16). Additional mechanisms include EBV-driven PD-L1 expression in subsets of lymphomas and leukemias, and chronic antigenic stimulation in the bone marrow niche promoting exhaustion (17).

In contrast to solid tumors, hematologic cancers often exhibit lower somatic mutational burden and fewer neoantigens, yet the amplified PD-L1 expression in specific subtypes overrides this limitation, explaining the outlier success in cHL/PMBCL (18). Preclinical models further demonstrate that PD-1 blockade enhances NK cell activity and reverses myeloid-derived suppressor cell suppression in the marrow microenvironment (19).

### 2.2 Evidence across major hematologic cancers

Classical Hodgkin lymphoma, PMBCL represents the flagship success of PD-1 blockade in hematologic oncology. Pivotal phase II trials established nivolumab (CheckMate 205) and pembrolizumab (KEYNOTE-087) as standards for r/r disease post-autologous transplant and brentuximab vedotin, with ORR 69–73%, complete response (CR) rates 16–29%, and median duration of response exceeding 16–24 months (16, 20). Long-term follow-up of CheckMate 205 (6–7 years) reported 5-year progression-free survival (PFS) with 72% in transplant-naïve patients and durable remissions beyond 5 years in ∼60% of responders (21). Pembrolizumab demonstrated 5-year OS ∼85% in KEYNOTE-087 updated analyses (22). These agents received FDA accelerated approval in 2016–2017 and full approval thereafter, transforming third-line management and now incorporated earlier (e.g., with AVD chemotherapy in the frontline) with 3-year PFS > 90% in phase III trials (23, 24).

Primary mediastinal large B-cell lymphoma shares the 9p24.1 alteration profile with cHL. Pembrolizumab in KEYNOTE-170 (r/r PMBCL) achieved ORR 45% (CR 13%) with durable responses (median DOR not reached at 3 + years), leading to FDA approval in 2018 (25). Nivolumab has shown similar activity (ORR ∼40–50%) in smaller series (26). PD-1 blockade is now guideline-preferred in r/r PMBCL.

#### 2.2.1 Non-Hodgkin lymphoma

In aggressive B-cell lymphomas, monotherapy yields modest results. Nivolumab in r/r Diffuse Large B-Cell Lymphoma (DLBCL) post-autologous transplant or ineligible for transplant showed ORR 10–36% in non-GCB subtypes, with CR rates < 10% (27). Pembrolizumab in KEYNOTE-013/170 achieved ORR ∼25% in Richter transformation but only 0–10% in standard DLBCL (28). Combination strategies (e.g., nivolumab + rituximab-gemcitabine-oxaliplatin) or with BTK inhibitors have improved ORR to 50–70% in early-phase studies (29, 30). Follicular lymphoma shows even lower activity (ORR < 10%) due to sparse PD-L1 expression and immunosuppressive follicular dendritic networks (31).

#### 2.2.2 T-cell lymphomas exhibit greater heterogeneity

Nivolumab/pembrolizumab monotherapy in r/r PTCL/NK-T-cell lymphoma yields ORR 20–40%, with higher rates in AITL or EBV-associated subtypes (32). Sintilimab and tislelizumab have shown ORR > 50% in relapsed NK/T-cell lymphoma in Asian cohorts (33). Overall, PD-1 blockade has limited single-agent approval in NHL but is increasingly combined with chemotherapy, bispecific antibodies, or lenalidomide in ongoing trials (7).

#### 2.2.3 MM (limited efficacy)

MM has proven largely refractory to PD-1/PD-L1 monotherapy, with ORR consistently < 10% across KEYNOTE and CheckMate trials (34, 35). Phase III trials combining pembrolizumab with lenalidomide-dexamethasone or pomalidomide-dexamethasone (KEYNOTE-183/185) were halted by the FDA in 2017 due to increased mortality in the experimental arms, attributed to excessive immune-related toxicity and lack of efficacy (36). Subsequent trials with nivolumab ± elotuzumab or pomalidomide also failed to show benefit (CheckMate 602, 2024) (37). Emerging data suggest modest activity when sequenced after BCMA-targeted therapies or combined with bispecific antibodies (talquetamab + cetrelimab ORR ∼60–70% in early reports), potentially by overcoming T-cell exhaustion post-BCMA redirection (38, 39).

#### 2.2.4 Leukemias (exceptions and ongoing trials)

Acute leukemias show minimal single-agent activity (ORR < 5% in AML). However, post-allogeneic HCT relapse represents an exception: nivolumab or pembrolizumab can induce durable complete remissions in 30–50% of AML/MDS patients via graft-versus-leukemia enhancement, with mixed chimerism emerging as a predictive factor (40, 41). Hypomethylating agents + PD-1 blockade trials show ORR 20–30% in frontline unfit AML, but no randomized superiority yet (42). Chronic lymphocytic leukemia has negligible responses.

### 2.3 Current predictive biomarkers and their limitations

PD-L1 expression by IHC (22C3 or 28-8 assays) is the most established biomarker in cHL/PMBCL, where 9p24.1 amplification correlates with near-universal expression and superior ORR/CR rates (> 80–90%) (9, 43). Tumor mutational burden is generally low (< 5 mut/Mb) in hematologic malignancies compared to MSI-high solid tumors, limiting its predictive value outside rare hypermutated cases (44). EBV status (LMP1-driven PD-L1) predicts response in NK/T-cell and PTCL (45).

These biomarkers fail in many hematological settings for several reasons: (i) low neoantigen load reduces baseline T-cell priming; (ii) dominant immunosuppressive marrow microenvironment [myeloid-derived suppressor cells (MDSCs), M2 macrophages, TGF-β] overrides PD-1 blockade; (iii) lack of standardized PD-L1 scoring in liquid tumors and assay discordance; (iv) confounding inflammation elevating PD-L1 without functional significance; (v) rapid resistance via alternative checkpoints (TIM-3, LAG-3, TIGIT) or MHC loss (46, 47). Composite scores incorporating soluble PD-L1, IFN-γ signature, or circulating tumor DNA are under investigation but not yet clinically implemented (48).

## 3 The immunologic role of endocrine and nutritional systems

The immune system does not operate in isolation but is profoundly shaped by systemic endocrine and nutritional signals that constitute a “whole-body immunologic tone” determining the baseline readiness of antitumor immunity (49, 50). This immuno-endocrine and immuno-nutritional crosstalk is bidirectional: immune activation feeds back to alter hormone secretion and nutrient partitioning, while endocrine/metabolic states reprogram immune cell fate, metabolism, and function (51). In cancer, chronic inflammation and tumor-derived factors frequently dysregulate these axes, fostering T-cell exhaustion, Treg/MDSC expansion, and impaired antigen presentation states that blunt the efficacy of PD-1/PD-L1 blockade (52). Emerging evidence across solid and hematologic malignancies demonstrates that pre-treatment endocrine–nutritional signatures represent modifiable host factors capable of predicting and potentially augmenting checkpoint inhibitor outcomes (10, 53).

### 3.1 Concept of immuno-endocrine and immuno-nutritional crosstalk

**FIGURE 1:**
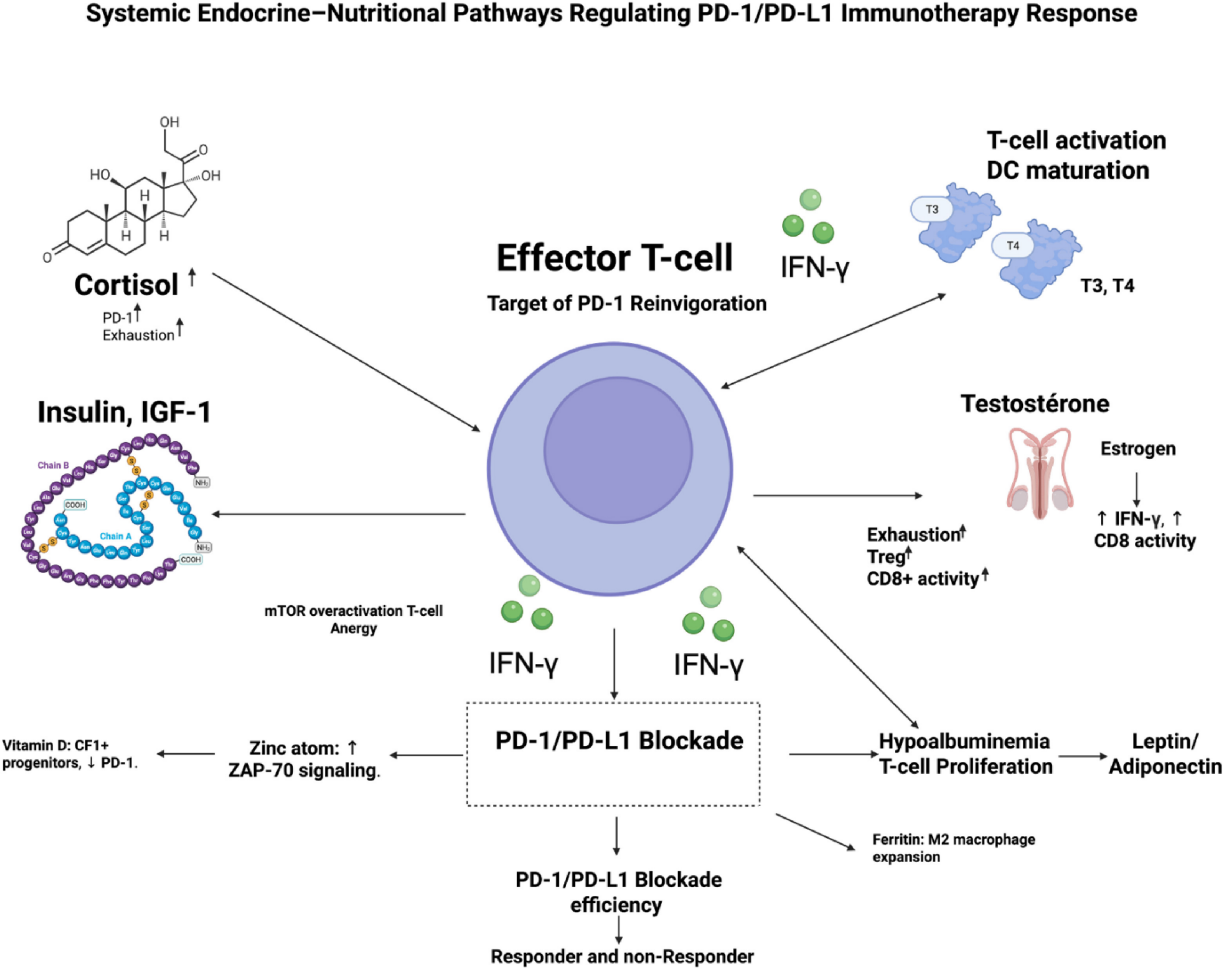
Mechanistic map of endocrine–nutritional pathways regulating PD-1/PD-L1 immunotherapy response. This schematic illustrates how systemic hormonal and nutritional signals converge on CD8^+^ effector T cells—the primary targets of PD-1 reinvigoration—to shape antitumor immunity. A central T cell displays PD-1 expression with IFN-γ–mediated tumor targeting. Surrounding endocrine regulators include: cortisol (adrenal axis), which drives PD-1 upregulation and T-cell exhaustion; thyroid hormones (T3/T4), enhancing T-cell activation and dendritic-cell maturation; insulin/IGF-1 signaling promoting mTOR overactivation and T-cell energy; and sex hormones, where estrogen supports IFN-γ production and CD8^+^ T cell cytotoxicity, whereas testosterone increases Treg activity and exhaustion. Nutritional cues include vitamin D maintenance of TCF1^+^ progenitors and reduced PD-1 levels, zinc enhancement of ZAP-70 signaling, iron overload/ferritin promoting M2 macrophage expansion, hypoalbuminemia impairing T-cell proliferation, and leptin–adiponectin imbalance skewing immune tone. These integrated inputs determine downstream PD-1/PD-L1 blockade efficacy, distinguishing potential responders from non-responders.

Immune cells express receptors for virtually all hormones, adipokines, and nutrient-sensing pathways (mTOR, AMPK, IGF-1R, VDR, AhR), allowing systemic metabolic cues to orchestrate leukocyte development, trafficking, and effector differentiation (54, 55). Conversely, cytokines (IFN-γ, IL-6, TNF-α) modulate hypothalamic–pituitary axes, adipocyte function, and nutrient transporter expression, creating feedback loops that can either amplify or suppress antitumor responses (50, 56). In the tumor microenvironment (TME), this crosstalk is hijacked: tumors induce chronic low-grade inflammation that elevates cortisol, leptin, and insulin while depleting micronutrients, driving T-cell dysfunction and resistance to PD-1/PD-L1 blockade (57). Preclinical models and clinical cohorts show that correcting these imbalances (e.g., vitamin D repletion, metformin-mediated insulin sensitization) restores CD8^+^ T-cell metabolism and synergizes with checkpoint inhibition (58, 59). The concept has particular relevance in hematologic malignancies, where marrow infiltration, cachexia, and prior therapies frequently induce profound endocrine–nutritional dysregulation (60). A mechanistic overview of how endocrine and nutritional pathways converge on T-cell immunity is shown in Figure 1.

### 3.2 Endocrine regulation of antitumor immunity

#### 3.2.1 Hypothalamic–pituitary–adrenal axis and cortisol

Chronic stress activates the HPA axis, elevating glucocorticoids (GCs) that potently suppress antitumor immunity. Endogenous and synthetic GCs upregulate PD-1 expression on CD8^+^ T cells via GR-mediated transactivation, accelerate exhaustion, impair proliferation, and promote apoptosis (61, 62). In tumor-bearing mice and patients, elevated cortisol or dexamethasone use correlates with reduced CD8^+^ T-cell infiltration, higher TIM-3/LAG-3 co-expression, and inferior response to PD-1 blockade (63, 64). Mechanistically, GCs inhibit mTORC1 signaling and glucose uptake in T cells while enhancing Treg suppressive function (65). Recent data show that tumor-intrinsic HSD11B1 reactivation of GCs limits IFN-γ signaling and ICI efficacy in melanoma (66).

#### 3.2.2 Thyroid hormones

Triiodothyronine (T3) and thyroxine (T4) enhance T-cell activation, dendritic cell (DC) maturation, and Th1 polarization via thyroid hormone receptor β expressed on immune cells (67). Subclinical or overt hypothyroidism, common in cancer patients, is associated with reduced CD8^+^ T-cell cytotoxicity and increased Treg frequency (68). Paradoxically, ICI-induced thyroiditis strongly predicts favorable outcomes across malignancies (ORR ↑ 2–3-fold), likely reflecting robust immune activation spilling into autoimmunity (69, 70). Low pre-treatment thyroid stimulating hormone (TSH) or free T4 correlates with poorer PFS/overall survival (OS) on PD-1 blockade, while thyroid hormone supplementation in hypothyroid models restores antitumor immunity (71).

#### 3.2.3 Sex hormones (estrogen, testosterone)

Sex hormones drive marked disparities in immunotherapy outcomes. Estrogen (via ERα/β) enhances CD8^+^ T cell effector function, DC cross-presentation, and IFN-γ production while reducing PD-1 expression, contributing to superior ICI responses in females in several cancers (72, 73). Conversely, testosterone suppresses Th1 responses, promotes Treg/MDSC accumulation, and upregulates PD-1/CTLA-4; androgen deprivation in prostate cancer models dramatically boosts ICI efficacy (74, 75). Large meta-analyses confirm male sex as an independent negative predictor of PD-1/PD-L1 benefit in prostate cancer/NSCLC, with hormonal aging (declining testosterone/estrogen) further exacerbating immune senescence (76, 77).

#### 3.2.4 Insulin/IGF-1 axis

Hyperinsulinemia and elevated IGF-1 signaling through PI3K/AKT/mTORC1 drives T-cell energy and exhaustion while promoting MDSC and M2 polarization (78). IGF-1R blockade or metformin in preclinical models reverses exhaustion, increases CD8^+^ T-cell infiltration, and synergizes with PD-1 inhibition (79, 80). Metabolic syndrome and high pre-treatment C-peptide predict inferior outcomes with ICIs, reflecting chronic inflammation and impaired T-cell metabolism (81).

#### 3.2.5 Adipokines (leptin, adiponectin)

Leptin, elevated in obesity, promotes T-cell exhaustion via mTOR activation and PD-1 upregulation while expanding Tregs; leptin-deficient mice show enhanced antitumor immunity (82). Conversely, adiponectin exerts anti-inflammatory effects, enhances CD8^+^ T-cell function, and correlates with better ICI responses (83). The obesity paradox improved ICI outcomes in overweight patients may partly reflect leptin-driven tonic signaling that paradoxically sustains effector T-cell survival during chronic stimulation (13, 84).

### 3.3 Nutritional regulation of immunity

#### 3.3.1 Protein status (albumin, prealbumin)

Hypoalbuminemia reflects chronic inflammation (IL-6-driven) and protein-energy malnutrition, impairing T-cell proliferation and cytokine production via reduced mTOR signaling and amino acid availability (85). Low albumin/prealbumin strongly predicts non-response to PD-1 blockade across cancers (86).

#### 3.3.2 Vitamins D, A, B12, folate

Vitamin D (via VDR on T cells/DC) promotes Th1/Tc1 differentiation, inhibits Treg, and enhances PD-L1 blockade efficacy; deficiency correlates with poorer survival and reduced CD8^+^ T-cell infiltration (12, 87). Preclinical and clinical data show vitamin D repletion overcomes resistance by remodeling the microbiome and boosting IFN-γ signaling (88, 89).

Vitamins A (retinoic acid), B12, and folate are essential for T-cell proliferation and thymic function; deficiencies common in hematologic patients impair DNA synthesis and cytotoxicity (90).

#### 3.3.3 Iron metabolism

Dysregulated iron handling (high ferritin, low transferrin) fuels MDSC and M2 macrophages while starving T cells of iron required for proliferation (91). Anemia and high hepcidin predict inferior ICI outcomes (92).

#### 3.3.4 Zinc, selenium, and trace elements

Zinc is critical for ZAP-70 signaling and NK/CD8^+^ T-cell cytotoxicity; deficiency increases PD-1^+^ exhausted T cells (93). Selenium (via selenoproteins) protects against oxidative stress during activation; low levels correlate with Treg expansion and reduced ICI benefit (93, 94).

### 3.4 Metabolic reprogramming of immune cells

#### 3.4.1 Amino acid metabolism (arginine, tryptophan)

Tumors and MDSCs deplete arginine (via ARG1) and tryptophan (via IDO1), inducing GCN2/mTOR inhibition, T-cell energy, and Treg differentiation (95, 96). IDO1 expression strongly predicts ICI resistance; inhibitors restore effector function in models (97).

#### 3.4.2 Lipid metabolic pathways

Effector T cells rely on fatty acid oxidation; obesity-associated hyperlipidemia paradoxically supports memory formation, but chronic cholesterol overload impairs TCR signaling via ER stress (98).

#### 3.4.3 Gut microbiome as a nutritional mediator

**FIGURE 2:**
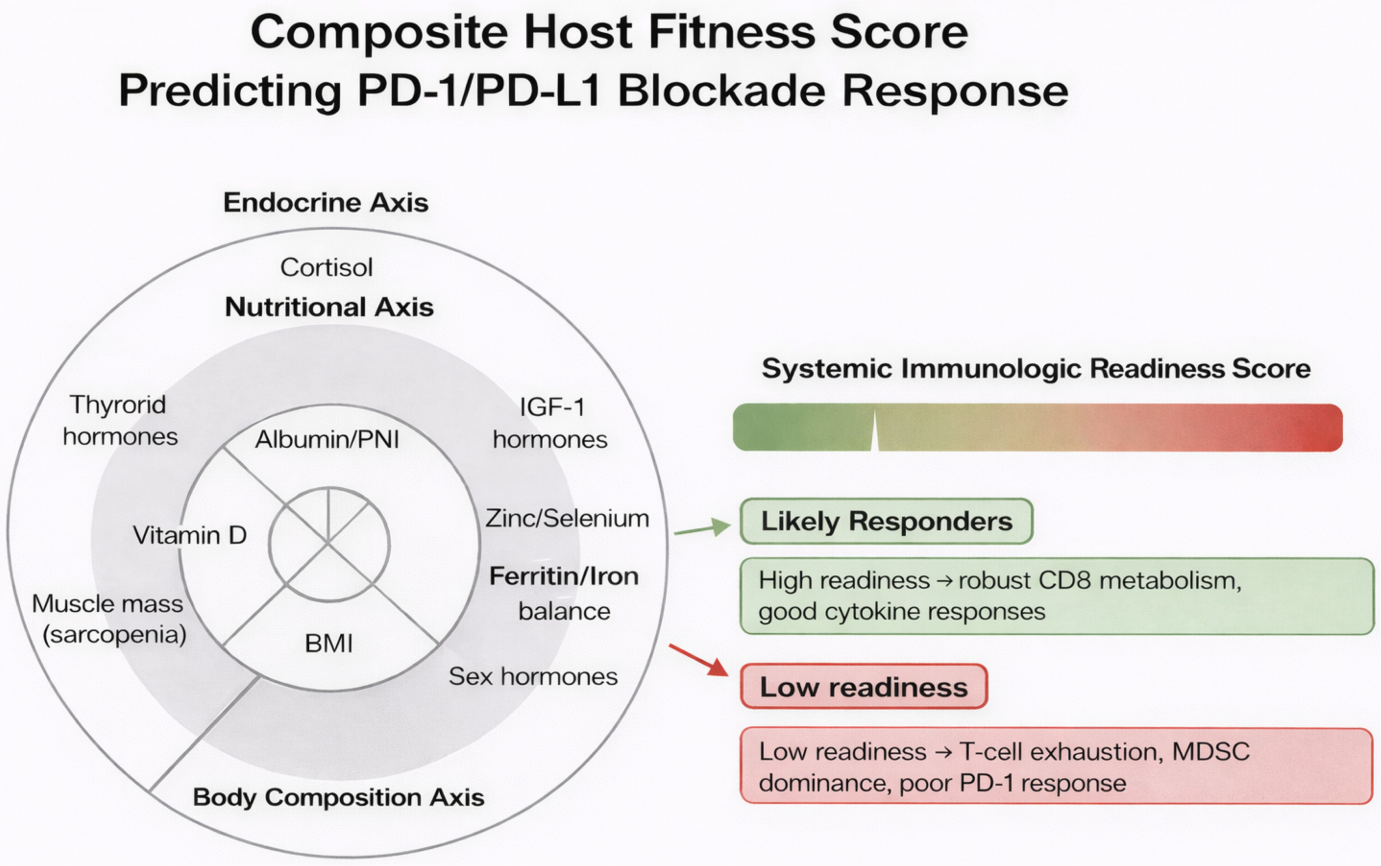
Integrated “immunologic readiness” scoring model for predicting PD-1/PD-L1 blockade response. This conceptual model aggregates endocrine, nutritional, and body composition domains to generate a composite *Systemic Immunologic Readiness Score*. A three-ring radar-style layout illustrates the contributing axes: (1) Endocrine Axis, encompassing cortisol levels, thyroid hormones, IGF-1, and sex hormones; (2) Nutritional Axis, capturing albumin/PNI, vitamin D, zinc–selenium status, and ferritin/iron balance; and (3) Body Composition Axis, reflecting muscle mass (sarcopenia status), fat mass/leptin activity, and BMI. These parameters integrate into a color-graded scoring meter (green→red) that stratifies patients as *likely responders* or *likely non-responders* to PD-1/PD-L1 therapy. Accompanying notes highlight the biological underpinnings: high readiness supports resilient CD8^+^ T-cell metabolism and strong cytokine production, whereas low readiness is associated with T-cell exhaustion, myeloid-derived suppressor cell (MDSC) dominance, and reduced checkpoint inhibitor efficacy.

Diet shapes microbiome composition, which systemically regulates ICI efficacy via microbial metabolites (SCFAs, inosine) that enhance DC maturation and CD8^+^ T-cell infiltration (99, 100). High-fiber diets enrich responder taxa (Akkermansia, Faecalibacterium) and improve ORR/PFS; Western diets deplete them and promote resistance (100, 101). These endocrine–nutritional determinants integrate into a composite immunologic readiness score. Figure 2 illustrates a conceptual framework linking host systemic physiology to response to PD-1/PD-L1 immune checkpoint blockade. The model integrates three interrelated biological axes—endocrine, nutritional, and body composition that collectively shape a composite host fitness score. Key biomarkers within these axes include cortisol and thyroid hormones (endocrine axis), IGF-1, albumin/PNI, micronutrient balance (zinc, selenium, ferritin/iron), and indicators of nutritional status (nutritional axis), as well as BMI and skeletal muscle mass reflecting sarcopenia (body composition axis). These parameters converge to define the System Immunologic Readiness Score, which represents the host’s capacity to mount an effective antitumor immune response. Patients with preserved metabolic and nutritional status, adequate muscle mass, and balanced endocrine signaling are positioned toward higher readiness, characterized by robust CD8^+^ T-cell metabolism and effective cytokine responses. In contrast, dysregulation across these axes such as sarcopenia, micronutrient deficiency, chronic stress signaling, or iron imbalance correlates with lower immunologic readiness, immune exhaustion, MDSC dominance, and diminished response to PD-1 blockade. Overall, the figure emphasizes that response to immune checkpoint inhibition is not solely tumor-intrinsic but is strongly influenced by systemic host factors, supporting the integration of metabolic, nutritional, and body composition assessments into immunotherapy stratification.

## 4 Evidence linking endocrine abnormalities to checkpoint inhibitor outcomes

Accumulating observational and mechanistic data demonstrate that pre-treatment endocrine abnormalities, particularly dysregulation of the HPA axis, thyroid function, sex hormones, insulin/IGF-1 signaling, vitamin D axis, and adipokine balance, significantly modulate clinical benefit from PD-1/PD-L1 blockade. These host factors influence baseline T-cell fitness, exhaustion state, and tumor microenvironment permissiveness, often explaining part of the heterogeneous responses observed across malignancies. Although most evidence derives from large cohorts in melanoma, NSCLC, and renal cell carcinoma, the immunologic mechanisms are conserved and increasingly corroborated in hematologic cancers (cHL, non-Hodgkin lymphomas, MM), where chronic inflammation and marrow niche effects amplify endocrine-immune crosstalk. Key endocrine biomarkers influencing PD-1/PD-L1 responses are summarized in Table 1.

**TABLE 1:** Endocrine biomarkers and their immunologic effects.

Endocrine marker	Immunologic effect/mechanism	References
Cortisol (glucocorticoids)	Broadly immunosuppressive. Increases PD-1 expression on T and NK cells, suppresses T-cell proliferation, and skews cytokine milieu (e.g., ↑IL-6). High baseline cortisol correlates with poor ICI response.	(102)
Thyroid hormones (T3/T4)	Modulate both innate and adaptive immunity. T3 drives dendritic cell maturation (↑MHC-II and costimulatory molecules) and pro-inflammatory cytokines (IL-12, IL-6, IL-23, IL-1β), promoting Th1/Th17 responses and reducing Tregs. T3-conditioned DCs also downregulate PD-L1 on DCs and PD-1 on T cells	(103)
Insulin	Immunomodulatory hormone. Immune cells express insulin receptors; insulin generally has anti-inflammatory effects and can modulate immune cell differentiation and polarization. It also enhances effector functions (e.g., ROS production by phagocytes).	(104)
IGF-1 (insulin-like growth factor 1)	Promotes immunosuppression. IGF-1 enhances FOXP3^+^ Treg function and IL-10 secretion (driving anti-inflammatory M2 macrophages), while suppressing antigen processing/presentation and upregulating PD-L1 on tumor/immune cells. High IGF-1 levels are linked to resistance to ICI.	(104)

### 4.1 Cortisol and stress-axis dysregulation

Chronic activation of the HPA axis and resultant hypercortisolism exerts profound systemic immunosuppression by directly inducing T-cell exhaustion and apoptosis while expanding immunosuppressive myeloid populations (105). Glucocorticoids (GCs) upregulate PD-1, CTLA-4, and TIM-3 on CD8^+^ T-cells via glucocorticoid receptor-mediated transactivation and transrepression of pro-inflammatory transcription factors (NF-κB, AP-1), simultaneously inhibiting IL-2 and IFN-γ production (61, 62).

Observational findings consistently link baseline GC use (≥ 10 mg prednisone equivalent daily, often for symptom control or comorbidities) with markedly inferior outcomes on PD-1/PD-L1 therapy. A landmark study in NSCLC showed that baseline corticosteroids were associated with reduced ORR (ORR: 8.5% vs. 32.3%), PFS (HR = 1.93), and OS (HR = 2.34) independent of performance status or brain metastases (106). Meta-analyses confirm this detrimental effect across tumor types, with dose- and duration-dependent impairment (107, 108). Endogenous hypercortisolism (e.g., Cushing syndrome or chronic stress-elevated morning cortisol > 500–600 nmol/L) similarly predicts resistance, as shown in preclinical models where tumor-derived GC reactivation via HSD11B1 limits IFN-γ signaling and CD8^+^ T -cell infiltration (66).

**FIGURE 3:**
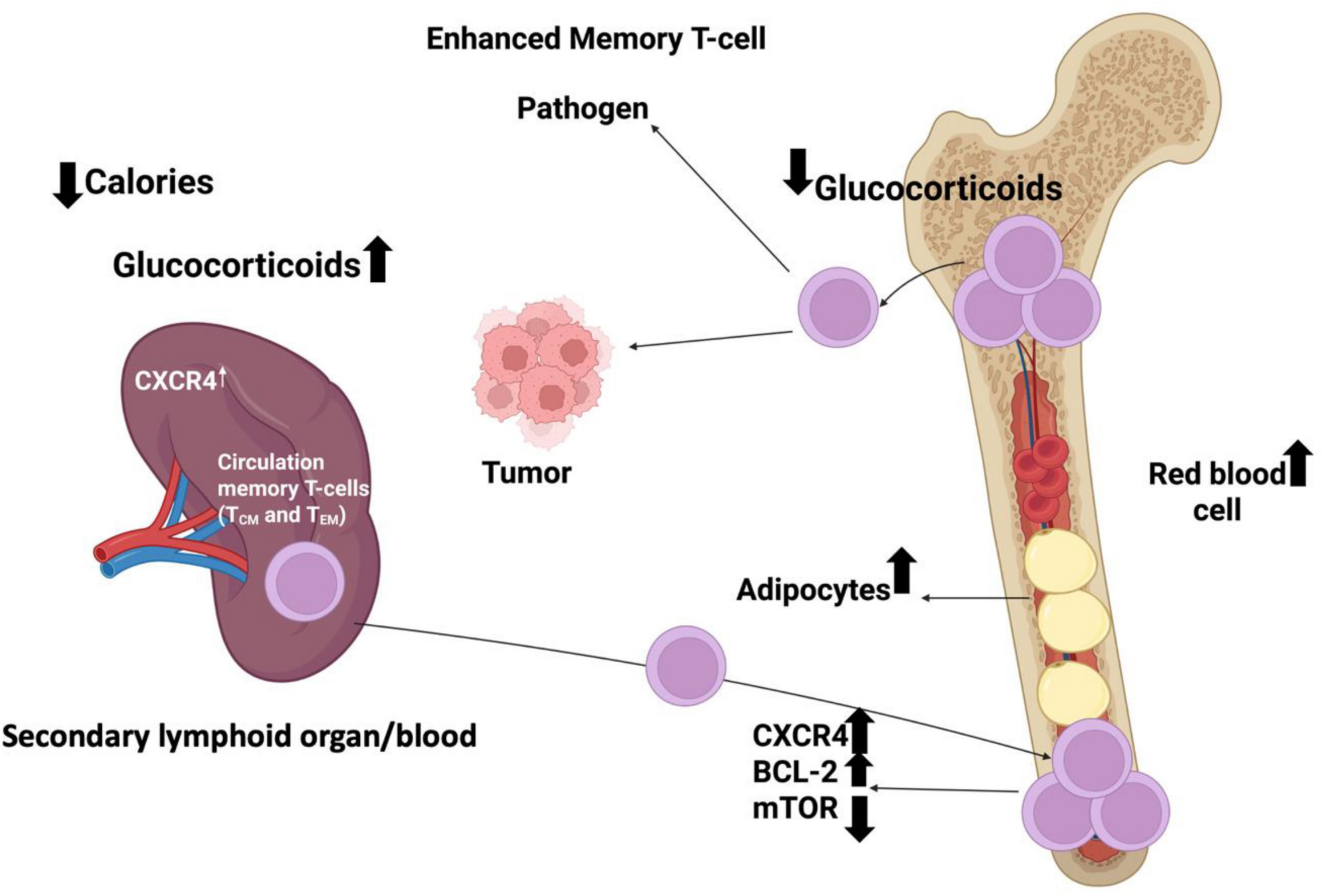
Schematic representation of how circulating memory T cells (T_CM and T_EM) redistribute between the spleen, blood, and bone marrow in response to tumor signals and corticosteroid treatment. Increased CXCR4 expression promotes the homing of circulating memory T cells to the bone marrow, where corticosteroids further enhance their retention. The diagram illustrates the movement of these T-cell subsets toward tumor tissue and their dynamic localization within hematopoietic and adipose niches of the bone marrow.

In hematologic malignancies, data are sparser but supportive: real-world cHL cohorts treated with nivolumab/pembrolizumab demonstrate shorter response duration in patients requiring GCs for symptom control or immune-related adverse events (irAEs) (5). Hypophysitis-induced secondary adrenal insufficiency (common irAE, incidence 5–15% with combination CTLA-4/PD-1 blockade) paradoxically does not worsen prognosis when promptly replaced with physiologic hydrocortisone, suggesting that supra-physiologic GC doses, not cortisol deficiency *per se*, drive immunosuppression (109, 110). The redistribution of circulating memory T cells under hypercortisolism and CXCR4 signaling is illustrated in Figure 3.

### 4.2 Thyroid dysfunction and T-cell activation

Thyroid hormones (T3/T4) are essential for T-cell development, proliferation, and Th1 polarization via thyroid receptor expression on lymphocytes and dendritic cells (67). PD-1/PD-L1 inhibitors frequently induce thyroid irAEs (irAE-thyroiditis, incidence 10–40%, highest with combination therapy), manifesting as transient thyrotoxicosis followed by hypothyroidism, reflecting destructive autoimmunity against thyroperoxidase/glutamate decarboxylase antibodies (69).

Multiple large cohorts and meta-analyses (> 10,000 patients) establish that development of thyroid dysfunction is a robust positive predictor of ICI benefit that compared to patients without thyroid irAEs (70, 111, 112). This association holds across melanoma, NSCLC, and renal cancer and appears independent of other irAEs, likely reflecting systemic immune activation spilling into thyroid autoimmunity (113). Mechanistically, ICI-induced thyroiditis correlates with increased CD8^+^ T-cell reinvigoration, reduced Treg frequency, and lower PD-1 expression on peripheral T cells (114).

**FIGURE 4:**
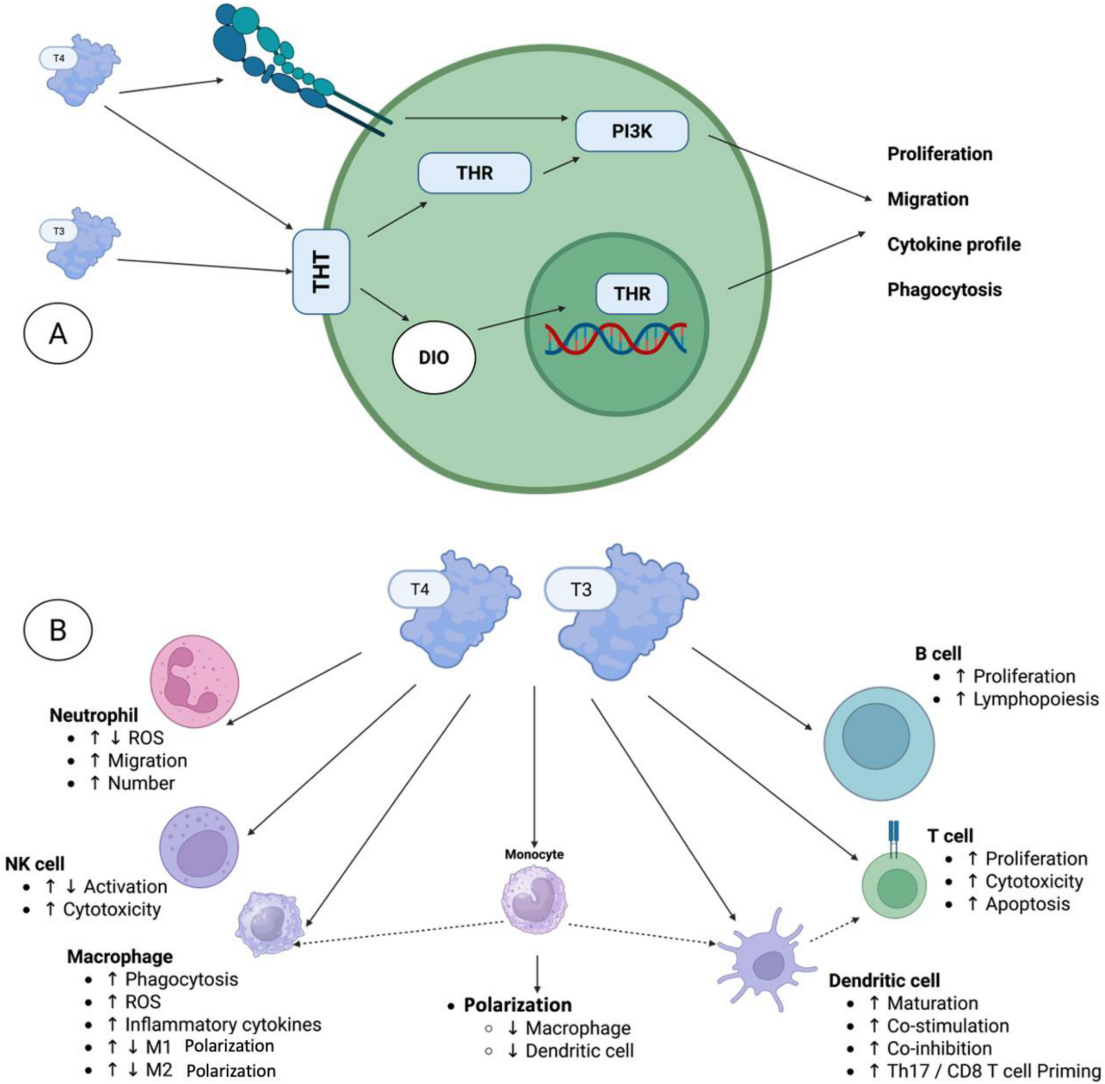
The local effects of thyroid hormones on the immune system. **(A)** Several immune cell types have been reported to express distinct thyroid hormone transporters (THTs), which mediate the uptake of thyroid hormones (THs) into the cells. Within the cell, deiodinases (DIOs) facilitate the conversion of thyroid hormones (THs), thereby either fostering or restricting TH activation. Intracellular T3 can subsequently bind to thyroid hormone receptors (THRs) in the cytoplasm or nucleus, thereby initiating non-canonical or canonical signaling pathways, respectively. In addition to the non-canonical thyroid hormone receptor action, which involves, among other mechanisms, PI3K signaling pathways, T4 can also bind to integrin αVβ3 on the cell surface, thereby initiating multiple pathways, including PI3K signaling. **(B)** The local effects of THs were observed in various innate and adaptive immune cells, including neutrophils, natural killer (NK) cells, macrophages, monocytes, dendritic cells, T cells, and B cells. Here, T3 and T4 were characterized as directly governing various functional processes, including activation, differentiation, proliferation, and/or migration. Furthermore, TH signaling in monocytes and dendritic cells indirectly influences the responses of macrophages and dendritic cells, respectively, as well as T cell activity.

Conversely, pre-existing hypothyroidism (often autoimmune Hashimoto’s) is associated with poorer outcomes, possibly via baseline Treg expansion and impaired T-cell priming (68). Subclinical hyperthyroidism or low TSH at baseline may also predict resistance in some series (115). In hematologic cancers, thyroid irAEs occur in 15–25% of cHL patients on PD-1 blockade and similarly predict prolonged responses, while untreated baseline hypothyroidism correlates with early progression in small DLBCL/MM cohorts (22, 31). The cell-specific effects of thyroid hormones on immune activation and differentiation are shown in Figure 4.

### 4.3 Sex hormones and immunotherapy response

Sex hormones profoundly shape antitumor immunity, with estrogen enhancing CD8^+^ T-cell effector function, dendritic cell cross-presentation, and IFN-γ signaling via ERα/β, while testosterone suppresses Th1 responses and promotes Treg/MDSC accumulation through androgen receptor signaling (72, 75).

A seminal 2018 meta-analysis of > 20 randomized trials (*n* > 11,000) found that males derive greater benefit from PD-1/PD-L1 inhibitors than females, a difference most pronounced in monotherapy versus chemotherapy controls (116). Subsequent analyses confirmed male advantage in melanoma, NSCLC, and head and neck cancer, with hazard ratios favoring males by 20–40% (76, 117). Mechanistically, androgen deprivation in prostate cancer models dramatically boosts CD8^+^ T-cell infiltration and synergizes with PD-1 blockade, while estrogen in females may upregulate alternative checkpoints (LAG-3, TIM-3) (118).

Hormonal aging exacerbates immune senescence: post-menopausal estrogen decline and age-related hypogonadism both correlate with reduced ICI efficacy (73). In hematologic malignancies, male sex is an adverse prognostic factor in some cHL real-world series treated with nivolumab (shorter PFS), consistent with testosterone-driven immunosuppression (119).

### 4.4 Insulin resistance and metabolic syndrome

Insulin resistance, type 2 diabetes, and metabolic syndrome drive chronic low-grade inflammation (elevated IL-6, TNF-α) that promotes T-cell exhaustion, MDSC expansion, and PD-L1 upregulation on tumor cells (78). Hyperinsulinemia activates PI3K/AKT/mTORC1 in T cells, inducing energy while impairing memory formation (79).

Retrospective studies and meta-analyses show that diabetes at baseline confers a 30–70% increased risk of progression or death on PD-1/PD-L1 therapy (HR 1.3–1.7), with hyperglycemia (> 200 mg/dL) during treatment independently predicting resistance (81, 120). Metabolic syndrome components (obesity + hypertension + dyslipidemia) compound this effect (121). Metformin, by ameliorating hyperinsulinemia and activating AMPK, has shown synergistic effects with ICIs in preclinical models and observational cohorts (improved ORR/PFS in diabetic patients) (122). Data in hematologic cancers are emerging: insulin resistance correlates with inferior responses in myeloma and DLBCL treated with PD-1 combinations (123).

### 4.5 Vitamin D endocrine axis

Vitamin D (via VDR on immune cells) promotes CD8^+^ T-cell activation, dendritic cell maturation, chemokine production (CXCL10), and gut microbiome diversity while inhibiting Treg and PD-1 expression (12, 124).

Multiple cohorts demonstrate that vitamin D deficiency (< 20 ng/mL) at baseline is associated with significantly worse ORR, PFS, and OS across ICI-treated cancers (125–127). A 2023 prospective study showed that systematic vitamin D supplementation (≥ 2,000 IU/day) increased ORR from 36 to 56% and median PFS from 5.8 to 11.3 months, with reduced severe irAEs (127). Genetic polymorphisms in VDR and CYP27B1 also predict outcomes (128). In hematologic cohorts, low vitamin D is prevalent (> 60% in lymphoma/MM) and correlates with poorer survival on PD-1-based regimens (129).

### 4.6 Adipokine imbalance as an immune checkpoint modulator

Adipose tissue secretes adipokines that bidirectionally regulate immunity: leptin promotes T-cell exhaustion via mTOR activation and PD-1 upregulation while expanding Tregs, whereas adiponectin exerts anti-inflammatory, CD8^+^ T-enhancing effects (82, 83).

Leptin levels are elevated in obesity and directly induce PD-1 on CD8^+^ T-cells in mouse models and human tumors, accelerating exhaustion; leptin signaling blockade restores ICI efficacy (82, 84). Yet a robust “metabolic obesity paradox” emerges: overweight/obese patients (BMI ≥ 25–30) consistently show superior ORR, PFS, and OS on PD-1/PD-L1 therapy across melanoma, NSCLC, and renal cancer in meta-analyses (> 30,000 patients) (13, 130). Proposed mechanisms include leptin-driven tonic signaling preventing terminal exhaustion, increased CD8^+^ T-cell infiltration, and altered pharmacokinetics (131). This paradox extends to hematologic malignancies: higher BMI predicts longer response duration in cHL treated with nivolumab/pembrolizumab in real-world series, though sarcopenic obesity negates the benefit (132, 133).

## 5 Nutritional and micronutrient profiles as predictors of PD-1/PD-L1 outcomes

Pre-treatment nutritional status and micronutrient profiles exert a profound influence on the efficacy of PD-1/PD-L1 blockade by directly modulating T-cell metabolism, proliferation, exhaustion state, antigen-presenting cell function, and the composition of the gut microbiome. In hematologic malignancies, where disease-related cachexia, chronic inflammation, marrow infiltration, and prior therapies frequently induce malnutrition, these host factors may be especially relevant to the heterogeneous responses observed with ICIs. While most robust evidence derives from cohorts of patients with solid tumors (melanoma, NSCLC, renal cell carcinoma, and gastrointestinal cancers the underlying immunologic mechanisms are shared and likely apply to lymphomas, MM, and leukemias. Emerging real-world data in cHL and diffuse large B-cell lymphoma (DLBCL) support similar trends. An overview of nutritional and micronutrient markers linked to checkpoint inhibitor efficacy is provided in Table 2.

**TABLE 2:** Nutritional and micronutrient markers linked to immunotherapy.

Nutritional/micronutrient marker	Role in ICI response/immune role	References
Vitamin D (25(OH)D)	Important immunomodulator (enhances innate immunity, regulates T-cell function). Higher baseline VitD correlates with better PD-1 responses and survival. VitD sufficiency is associated with higher response rates and fewer severe irAEs.	(134)
Zinc	Essential for thymic function and T-cell development. Adequate zinc supports T-cell and NK-cell function. High serum zinc predicts improved ICI outcomes (↑OS), possibly via NF-κB/MAPK pathways. Zinc deficiency impairs antitumor immunity.	(127)
Body mass index (BMI)	General nutritional status. In solid tumors, obesity often paradoxically correlates with better ICI outcomes (“obesity paradox”), but in hematologic malignancies, this is not consistent. In one cHL study, BMI (under/overweight vs. normal) did not affect nivolumab PFS/OS	(119)
Albumin	Marker of nutrition/inflammation. Hypoalbuminemia (malnutrition/inflammation) strongly predicts poor ICI outcomes. Pretreatment low albumin (e.g. < lower limit) was associated with much worse OS/PFS (HR ∼4) in melanoma. High albumin is linked to better survival on PD-1 therapy.	(119)
Ferritin (iron storage)	Acute-phase reactant and iron store. High ferritin often reflects inflammation and has been reported as a poor prognostic factor in ICI-treated patients (e.g., in lung cancer, high ferritin predicted worse outcomes). (Mechanism: may indicate chronic inflammation or immune exhaustion.)	(135)

### 5.1 Protein-energy malnutrition and survival

Protein-energy malnutrition (PEM) is highly prevalent in hematologic malignancies, affecting 30–60% of patients with aggressive lymphomas and MM, and is characterized by involuntary weight loss, hypoalbuminemia (<35 g/L), low prealbumin (<20 mg/dL), and reduced lean body mass. Multiple large retrospective cohorts have demonstrated that low baseline serum albumin and low Prognostic Nutritional Index (PNI) (PNI = 10 × albumin [g/dL] + 0.005 × lymphocyte count [/mm^3^]) are independent predictors of inferior objective response rate (ORR, PFS), and OS in patients receiving anti-PD-1/PD-L1 therapy (13, 82, 130, 136).

Mechanistically, hypoalbuminemia reflects chronic systemic inflammation (elevated IL-6, TNF-α) that drives T-cell exhaustion through persistent PD-1 upregulation and reduced mTOR pathway suppression, limiting the amino acid availability required for effector T-cell expansion and memory formation (137). In a multicenter Italian study of > 1 000 patients treated with anti-PD-1/PD-L1 agents, albumin < 35 g/L conferred a hazard ratio of 1.92 for death (82). Similar findings have been reported with the PNI, where PNI < 45 predicted significantly lower ORR (≈15–20% vs. 40–50% in high PNI) and shorter median OS in NSCLC and other cancers (136, 138).

In hematologic malignancies, low albumin is incorporated into established prognostic scores (e.g., International Prognostic Score for Hodgkin lymphoma, R-IPI for DLBCL) and retains prognostic significance in the immunotherapy era. Real-world cohorts of relapsed/refractory cHL treated with nivolumab or pembrolizumab have shown that albumin < 35 g/L is associated with markedly shorter duration of response and increased risk of early progression (139, 140). Prealbumin may be even more sensitive, reflecting acute changes in visceral protein status and predicting non-response in a small series of myeloma patients receiving PD-1-based combinations (141). These data strongly suggest that routine pre-treatment nutritional screening with albumin or PNI could identify patients requiring aggressive supportive care to optimize immunotherapy benefit.

### 5.2 Iron metabolism, anemia, and immunotherapy response

Anemia affects up to 70% of patients with lymphoma or myeloma at diagnosis and is exacerbated by disease progression or prior therapies. Baseline anemia (Hb < 10–11 g/dL) consistently predicts poorer outcomes with PD-1/PD-L1 blockade across tumor types, with meta-analyses showing hazard ratios of 1.5–2.0 for death (99, 100). High serum ferritin (> 300–500 ng/mL, context-dependent) as an acute-phase reactant reflects systemic inflammation and correlates with resistance to checkpoint inhibition, increased risk of hyperprogressive disease (142, 143).

Mechanistically, elevated hepcidin in inflammatory states sequesters iron in macrophages, depriving T cells of the iron required for proliferation and effector differentiation while simultaneously promoting M2-polarized immunosuppressive macrophages and myeloid-derived suppressor cells (144). In NSCLC cohorts, ferritin > 400 ng/mL was independently associated with reduced OS (HR 1.6–2.1) and lower ORR (145). In hematologic malignancies, hyperferritinemia is a well-known adverse factor in cHL and DLBCL and likely contributes to the immunosuppressive marrow microenvironment that limits PD-1 blockade efficacy in myeloma or leukemia (131). Careful correction of anemia (preferably with transfusion or erythropoietin-sparing approaches) and avoidance of iron overload may represent low-risk interventions to improve immunotherapy outcomes.

### 5.3 Micronutrients and antioxidant trace elements

Zinc and selenium deficiencies are frequent in hematologic malignancies due to poor intake, malabsorption, and increased utilization. Zinc is essential for thymic function, ZAP-70 signaling, and cytotoxic T-cell activity; low serum zinc levels (< 70 μg/dL) have been associated with significantly lower response rates and shorter PFS/OS in NSCLC patients receiving PD-1 inhibitors (84, 93). Selenium, incorporated into selenoproteins (e.g., GPx-4), protects T cells from oxidative stress during chronic antigen stimulation; low selenium status correlates with higher Treg frequency and reduced effector T-cell function in DLBCL and other cancers (146).

Supplementation studies, though limited, suggest that restoring zinc or selenium levels can enhance CD8^+^ T-cell cytotoxicity and synergize with PD-1 blockade in preclinical models (92). In DLBCL, low selenium was associated with increased PD-1^+^ Treg populations, suggesting a direct mechanism of immune evasion that could be reversed by supplementation (93). Routine micronutrient screening and correction may therefore represent a simple, low-cost strategy to augment checkpoint inhibitor efficacy in hematologic patients.

### 5.4 Diet patterns and metabolomics signatures

Dietary patterns profoundly shape the gut microbiome, which in turn systemically modulates response to PD-1/PD-L1 blockade. High-fiber, plant-rich diets are associated with enrichment of responder-associated taxa (Akkermansia muciniphila, Faecalibacterium prausnitzii, *Bifidobacterium* spp.) and significantly higher ORR and PFS in melanoma and NSCLC patients treated with anti-PD-1 therapy (147, 148). Conversely, Western-style high-fat/processed food diets correlate with unfavorable microbiome composition and reduced benefit (147). Metabolomic profiling has identified pre-treatment elevations in short-chain fatty acids (butyrate, propionate) and certain amino acid metabolites as predictors of response, reflecting microbial fermentation products that enhance dendritic cell function and T-cell infiltration (132).

Although direct evidence in hematologic malignancies is limited, dysbiosis is common in lymphoma and myeloma patients (often exacerbated by antibiotics or chemotherapy), and the gut microbiome’s influence on systemic immunity and GVHD after transplant suggests similar relevance for checkpoint inhibitor efficacy (149). Dietary interventions promoting favorable microbiome composition represent a promising adjunctive strategy.

### 5.5 Obesity, sarcopenia, and body composition analyses

An “obesity paradox” has been repeatedly demonstrated in immunotherapy, with overweight/obese patients (BMI ≥ 25 or ≥ 30 kg/m^2^) exhibiting superior ORR (up to 2-fold higher), PFS, and OS compared to normal-weight patients in large multicenter cohorts and meta-analyses. The effect is particularly striking in melanoma, NSCLC, and renal cell carcinoma, with hazard ratios for death of 0.6–0.7 in obese patients (150, 151). Proposed mechanisms include increased leptin signaling enhancing CD8^+^ T-cell function, greater energy reserves supporting prolonged immune activation, and altered adipokine profiles favoring Th1 polarization (152). Preliminary data in classical Hodgkin lymphoma treated with PD-1 inhibitors also suggest a similar trend, with higher BMI associated with improved response duration (153). Representative clinical studies examining endocrine and nutritional predictors of ICI outcomes are summarized in Table 3.

**TABLE 3:** Studies evaluating endocrine/nutritional predictors of ICI outcomes.

Study characteristics	Biomarker(s) analyzed	Key findings	References
(Retrospective cohort; 133 patients with relapsed/refractory classical Hodgkin lymphoma on nivolumab)	BMI categories (underweight, normal, overweight, obese)	No significant association between BMI and ICI efficacy: PFS/OS and response rates were similar across BMI groups; underweight individuals had worse outcomes, but trends were non-significant. BMI did *not* predict irAE risk in cHL.	(119)
(Prospective cohort; 77 advanced non-small cell lung cancer patients on PD-1/PD-L1 inhibitors)	Serum 25(OH) vitamin D	Higher baseline VitD was independently associated with better objective response (PR rate) and longer survival. Patients with 25(OH)D > 15.7 ng/mL had ∼3 × higher odds of response (OR 2.93) and improved OS. VitD insufficiency predicted poorer ICI benefit.	(119)
(Retrospective study; 98 advanced/metastatic cancer patients [lung, esophageal, gastric, colorectal] on ICIs)	Serum zinc level	Elevated baseline zinc (> 14.2 μg/L) strongly predicted improved OS and clinical benefit on PD-1/L1 therapy. Patients with higher zinc had longer OS (20 vs. 10 months). Zinc was proposed as a novel positive biomarker, potentially via modulating NF-κB/MAPK immune pathways.	(93)
(Retrospective multi-omic; metastatic melanoma patients on anti-PD-1 ± CTLA-4)	Serum albumin	Pretreatment hypoalbuminemia emerged as the strongest predictor of poor outcome. Low albumin (< LLN) was associated with much worse OS (HR∼4.0) and PFS (HR∼3.7). Even after adjusting for LDH, IFN-γ signature, TMB, etc. Normal albumin predicted durable benefit.	(93)
(Retrospective; 179 mixed cancer patients on ICIs)	Albumin–globulin ratio (AGR)	Lower AGR (< 1.21) (reflecting low albumin/high inflammation) was associated with significantly reduced OS (HR 1.53) and PFS (HR 1.39) on ICI therapy. AGR may capture combined nutritional/inflammatory status predicting ICI outcomes.	(135)

## 6 Integrated endocrine–nutritional signatures: a new predictive paradigm

Single biomarkers (e.g., albumin, vitamin D, BMI) frequently fail to reliably predict PD-1/PD-L1 blockade outcomes due to biological redundancy, confounding by disease-related inflammation, and limited capture of multifaceted immuno-endocrine-nutritional crosstalk. Integrated multi-marker signatures combining inflammatory (CRP, NLR), nutritional (albumin, PNI), metabolic (vitamin D, sarcopenia), and adipokine measures offer superior prognostic stratification across cancers treated with ICIs. These composite scores reflect a holistic “systemic immunologic readiness profile” that gauged host capacity to mount and sustain antitumor T-cell responses. Systemic immunologic readiness is the integrated host physiological state determined by endocrine balance, nutritional status, metabolic health, and systemic inflammation that collectively shapes baseline immune competence and the capacity of T cells to be reinvigorated by PD-1/PD-L1 blockade. This concept reflects a whole-body immune context that exists before therapy and modulates treatment responsiveness independently of tumor-intrinsic features. Although most evidence stems from solid tumors, translational data in hematologic malignancies (particularly cHL, diffuse large B-cell lymphoma [DLBCL], and MM) support applicability, with unique marrow-driven metabolic features warranting tailored panels (154). The integrated endocrine–nutritional signatures proposed for hematologic malignancies are summarized in Table 4.

**TABLE 4:** Proposed multi-marker signature for hematologic malignancies.

Signature components	Rationale/interactions	Supporting evidence	References
“Stress–Inflammation” signature: High cortisol + ↑IL-6 + low albumin	HPA axis activation + malnutrition/inflammation. Elevated cortisol (stress) drives immunosuppression (up PD-1) and systemic inflammation (↑IL-6). Low albumin reflects a poor nutritional/inflammatory state. Together, this profile indicates an immunosuppressed, catabolic state unlikely to respond to ICI.	Cortisol↔poor PFS/OS; Cortisol↑ linked to ↑IL-6; Hypoalbumin↔poor ICI outcome.	(102)
“Thyroid-DC activation” signature: High T3 (active TH signaling) + low PD-1/PD-L1 checkpoint	Enhanced antigen presentation and adaptive immunity. High T3 promotes dendritic cell maturation and pro-inflammatory cytokine production (IL-12, IL-6, IL-1β), driving Th1/Th17 and cytotoxic T-cell responses. T3 conditions DCs to downregulate PD-L1 and reduce PD-1 on T cells, lowering immune checkpoints. This endocrine-driven signature suggests a more responsive immune milieu.	T3-DC axis yields proinflammatory Th1/Th17 bias\ and lowers PD-1/PD-L1 in DC-T interactions.	(135)
“Micronutrient sufficiency” signature: High vitamin D + high zinc (+ normal albumin)	Supportive immune milieu. Adequate VitD and zinc are both required for effective innate and adaptive immunity. Sufficient VitD enhances T-cell regulation and anti-tumor immunity. High zinc supports T-cell/NK function and promotes favorable NF-κB signaling. Together (with normal albumin), this signature implies good nutritional status and immune competence, correlating with robust ICI response.	Baseline VitD sufficiency is linked to higher ICI ORR/OS; High zinc predicts longer ICI survival.	(103, 127)
“IGF/metabolic” signature: High IGF-1 (± high insulin or obesity)	Tumor immune evasion. Elevated IGF-1 drives regulatory/Treg-mediated immunosuppression and impairs antigen presentation. It upregulates PD-L1 and downregulates antigen-processing machinery in tumor cells. In a multi-marker model, high IGF-1 (often in metabolic syndrome) may identify patients with TME resistant to PD-1 blockade.	IGF-1 promotes FOXP3∧ + Tregs and IL-10; IGF-1 upregulates PD-L1/downregulates antigen presentation.	(93)

### 6.1 Why single biomarkers are insufficient

Individual endocrine or nutritional markers demonstrate inconsistent predictive performance for ICI efficacy due to high inter-patient variability, overlapping confounders (comorbidities, steroids, cachexia), and inability to capture synergistic interactions across immuno-metabolic pathways (155, 156). For example, while hypoalbuminemia robustly predicts inferior outcomes, its specificity is limited by non-nutritional causes (hepatic dysfunction, nephrotic syndrome) and failure to account for compensatory mechanisms like adipokine signaling in obesity (85). Similarly, vitamin D deficiency correlates with resistance but shows heterogeneous effect sizes across cohorts, modulated by baseline inflammation or microbiome status (157). BMI alone yields paradoxical results (obesity benefit in many settings) yet ignores muscle quality, where sarcopenia independently drives exhaustion (82). Meta-analyses confirm modest hazard ratios (1.3–1.8) for single markers versus > 2.5–4.0 for composites, underscoring the need for integrated approaches that better reflect the complex “whole-body immunologic tone” governing T-cell fitness (136, 137). Table 5 synthesizes the current clinical evidence linking endocrine and nutritional parameters with PFS and OS in cancer patients receiving ICIs. Collectively, the studies summarized in this table highlight that host metabolic and hormonal status is not merely a background variable but a biologically active determinant of immunotherapy efficacy. Across multiple tumor types and treatment settings, endocrine alterations such as dysregulated thyroid function, cortisol excess, insulin resistance, and sex hormone imbalance emerge as modulators of antitumor immune responses, influencing T-cell activation, exhaustion dynamics, and immune-related adverse event profiles. In parallel, nutritional indices including BMI, sarcopenia, cachexia, serum albumin, PNI, and inflammatory-nutritional composites (e.g., CONUT, GPS, NLR-based scores) consistently correlate with survival outcomes, underscoring the role of systemic energy availability and muscle–immune crosstalk in sustaining effective immune surveillance. Notably, several studies report paradoxical findings such as improved outcomes in overweight patients supporting the concept of an “immunometabolic reserve” that may buffer immune cells against ICI-induced metabolic stress.

**TABLE 5:** Summary of key studies evaluating endocrine-nutritional predictors and their associations with progression-free survival (PFS) and overall survival (OS) in patients treated with immune checkpoint inhibitors (ICIs).

Author references	Cancer type	Predictor	HR for PFS (95% CI)	HR for OS (95% CI)
Li et al. (158)	Advanced cancers (including some Hodgkin lymphoma) on PD-1/PD-L1 inhibitors	Low PNI (vs. high)	1.75 (1.40–2.18)	2.31 (1.81–2.94)
Jiang et al. (159)	Hematological malignancies (general, not ICI-specific)	High GPS (vs. low)	∼2.0 (exact CI not specified in abstract)	∼2.0 (exact CI not specified in abstract)
Bersanelli et al. (125)	Advanced solid tumors on ICIs	Vitamin D supplementation (vs. none)	0.61 (0.40–0.91) for TTF	0.55 (0.34–0.90)
Guo et al. (160)	Lung cancer (NSCLC and SCLC) on ICIs	Presence of irTD (vs. absence)	Not significant overall	0.65 (0.49–0.88)
Kopanos et al. (161)	Melanoma, NSCLC, urothelial cancer on ICIs	Presence of endocrine irAEs (vs. absence)	0.61 (0.54–0.68)	0.60 (0.54–0.67)
Witte et al. (162)	Multiple myeloma (general, not ICI-specific)	Higher GPS (vs. lower)	1.405 (1.058–1.867)	2.127 (1.431–3.162)
Shi et al. (163)	Advanced NSCLC on immunotherapy	High PNI (> 45 vs. ≤ 45)	0.405 (0.184–0.892)	0.294 (0.123–0.703)

While the search focused on hematologic malignancies and ICI, many studies are from solid tumors or general hematologic contexts due to limited specific data. Endocrine-nutritional predictors like PNI and GPS show consistent prognostic value across cancers, with low values indicating worse outcomes. For hematologic-specific ICI, data are emerging but sparse; e.g., PNI included Hodgkin lymphoma in mixed cohorts. Further research is needed for targeted hematologic applications.

### 6.2 Concept of a “systemic immunologic readiness profile”

The systemic immunologic readiness profile conceptualizes pre-treatment host status as a composite continuum from “fit” (optimal nutrient reserves, balanced hormones, low inflammation supporting robust CD8^+^ T cell reinvigoration) to “unfit” (malnutrition, endocrine dysregulation, chronic inflammation enforcing exhaustion and Treg/MDSC dominance) (51, 164). This paradigm shifts focus from tumor-intrinsic features (PD-L1, TMB) to modifiable host factors that determine whether PD-1/PD-L1 blockade can restore effective immunity. Preclinical models demonstrate that combined insults (e.g., cortisol elevation + zinc deficiency + arginine depletion) synergistically impair mTOR signaling and IFN-γ production beyond any single factor (57). Clinically, patients with “fit profiles exhibit higher ORR (50–80% vs. < 20%), longer PFS/OS, and fewer severe irAEs, likely via enhanced T-cell metabolism and reduced alternative checkpoint upregulation (165). In hematologic malignancies, where systemic disease and prior therapies amplify host dysregulation, this profile may explain outlier successes (cHL) versus failures (MM monotherapy) (166).

### 6.3 Multi-marker signatures studied to date

#### 6.3.1 Albumin + CRP indices

The Glasgow Prognostic Score (GPS) (GPS: CRP > 10 mg/L and albumin < 35 g/L) and modified GPS (mGPS) are among the most validated composites, integrating acute-phase response with visceral protein status. In metastatic cancers treated with ICIs, high GPS/mGPS independently predicts poorer ORR, PFS, and OS across melanoma, NSCLC, and RCC in large cohorts and meta-analyses (167–169). The PNI (PNI = 10 × albumin + 0.005 × lymphocytes) similarly outperforms single markers, with low PNI (< 45) associated with HR 2.0–3.5 for progression in ICI-treated gastric, lung, and mixed cancers (138, 139). Controlling Nutritional Status (CONUT) score adds cholesterol, further refining risk stratification (170).

#### 6.3.2 Vitamin D + inflammatory markers

Combining vitamin D (< 20 ng/mL deficiency) with high CRP, NLR (> 3–5), or GPS enhances predictive accuracy. In NSCLC and melanoma cohorts, deficient vitamin D + elevated inflammation confers the worst outcomes (median PFS < 4 months vs. > 24 months in sufficient/low-inflammation), reflecting impaired DC maturation and microbiome dysbiosis (127, 171). Prospective trials of vitamin D supplementation in high-risk (inflammatory) patients show ORR improvement from ∼35 to ∼55%, supporting dynamic interplay (172).

#### 6.3.3 Sarcopenia + adipokine levels

Body composition analyses reveal sarcopenic obesity (low muscle + high fat) as a high-risk phenotype, combining leptin-driven exhaustion with reduced myokine support for T cells. Meta-analyses (> 5,000 ICI patients) report HR 2.0–3.5 for death in sarcopenic obesity vs. obesity alone (protective HR 0.6–0.7), with leptin/adiponectin ratio emerging as a mechanistic link (173–175). Low adiponectin + sarcopenia predicts severe irAEs and resistance, while high leptin in non-sarcopenic obesity correlates with benefit (83).

### 6.4 Translating these signatures to hematologic cancers

Hematologic malignancies exhibit unique metabolic vulnerabilities: aggressive lymphomas and myeloma frequently induce protein-energy wasting (30–60% prevalence), hypercatabolism, and marrow adipose tissue remodeling that amplifies MDSC and impair hematopoiesis (19, 176). In cHL, the obesity paradox is pronounced higher BMI/leptin associates with superior duration of response to nivolumab/pembrolizumab, possibly via sustained CD8^+^ T-cell metabolism overriding 9p24.1-driven evasion (119). DLBCL shows similar trends, with low PNI/GPS predicting early relapse post-ICI combinations (177). MM’s refractory nature to PD-1 monotherapy may stem from profound cachexia, vitamin D deficiency (bone disease), and IGF-1 dysregulation in the marrow niche, fostering T-cell energy (178).

The marrow microenvironment rich in adipocytes, cytokines (IL-6), and hepcidin—amplifies endocrine-nutritional effects: leptin from marrow fat promotes Treg, while iron dysregulation starves T cells (179). Real-world cHL series incorporating PNI report low scores in 40–50% of relapsed patients, correlating with reduced CR rates (139). Emerging data in CAR-T (analogous immune activation) validate CONUT/PNI as predictors in MM, suggesting relevance for ICI combinations (180).

### 6.5 Potential for composite pre-treatment predictive panels

Composite panels integrating 3–6 markers (e.g., PNI/GPS + vitamin D + sarcopenia index + leptin/adiponectin ratio ± cortisol) could achieve > 80% accuracy in stratifying ICI responders, enabling precision supportive care (nutrition repletion, exercise, metformin, vitamin D) to convert “unfit” to “fit” hosts (180, 181). Machine learning-derived scores (e.g., combining SII + CONUT + BMI) outperform traditional tools in solid tumors and are being validated in lymphoma trials. In hematologic settings, proposed panels might add marrow-specific factors (e.g., ferritin, free light chains) for personalized prediction, potentially augmenting low baseline efficacy in MM or leukemias (182). Prospective trials are essential to standardize cut-offs, timing (circadian variability in cortisol), and interventions.

## 7 Mechanistic insights: how endocrine and nutritional status alter immune checkpoint response

Endocrine and nutritional status profoundly reprograms immune cell metabolism, signaling, and gene expression, thereby modulating the depth and durability of response to PD-1/PD-L1 blockade. Dysregulated states (hypercortisolism, insulin resistance, leptin excess, vitamin D/zinc deficiency, arginine/tryptophan depletion) converge on pathways that accelerate T-cell exhaustion, suppress antigen presentation, enrich immunosuppressive myeloid cells, and blunt cytokine-driven reinvigoration mechanisms that directly undermine checkpoint inhibitor efficacy (11, 55, 183). Conversely, optimal endocrine–nutritional balance sustains effector T-cell metabolism (glycolysis, OXPHOS, fatty acid oxidation), prevents terminal exhaustion, and enhances IFN-γ-dependent PD-L1 upregulation on tumors, creating a permissive milieu for PD-1/PD-L1 blockade (58, 184). These effects are amplified in hematologic malignancies by the bone marrow niche, where adipocytes, cytokines, and nutrient competition impose additional metabolic constraints (179).

### 7.1 Impact on T-cell exhaustion, proliferation, and memory

T-cell exhaustion, a hypofunctional state characterized by hierarchical loss of effector cytokines, high co-inhibitory receptor expression (PD-1, TIM-3, LAG-3), and epigenetic remodeling, is metabolically regulated (185). Glucocorticoids, via GR-mediated transactivation, directly upregulate PD-1 and TOX/TOX2 transcription factors while inhibiting mTORC1 and glucose uptake, locking CD8^+^ T-cells in exhaustion and preventing proliferation/memory formation; blockade of GC signaling restores ICI responsiveness in preclinical models (61, 66).

Leptin, elevated in obesity, activates STAT3/mTORC1 in T cells, leptin-neutralization reinvigorates exhausted T cells and synergizes with PD-1 blockade (82, 84). Hyperinsulinemia/IGF-1 similarly hyperactivates PI3K/AKT/mTORC1, inducing exhaustion while impairing CD8^+^ memory T precursors; metformin-mediated AMPK activation reverses this and boosts ICI efficacy (186).

**FIGURE 5:**
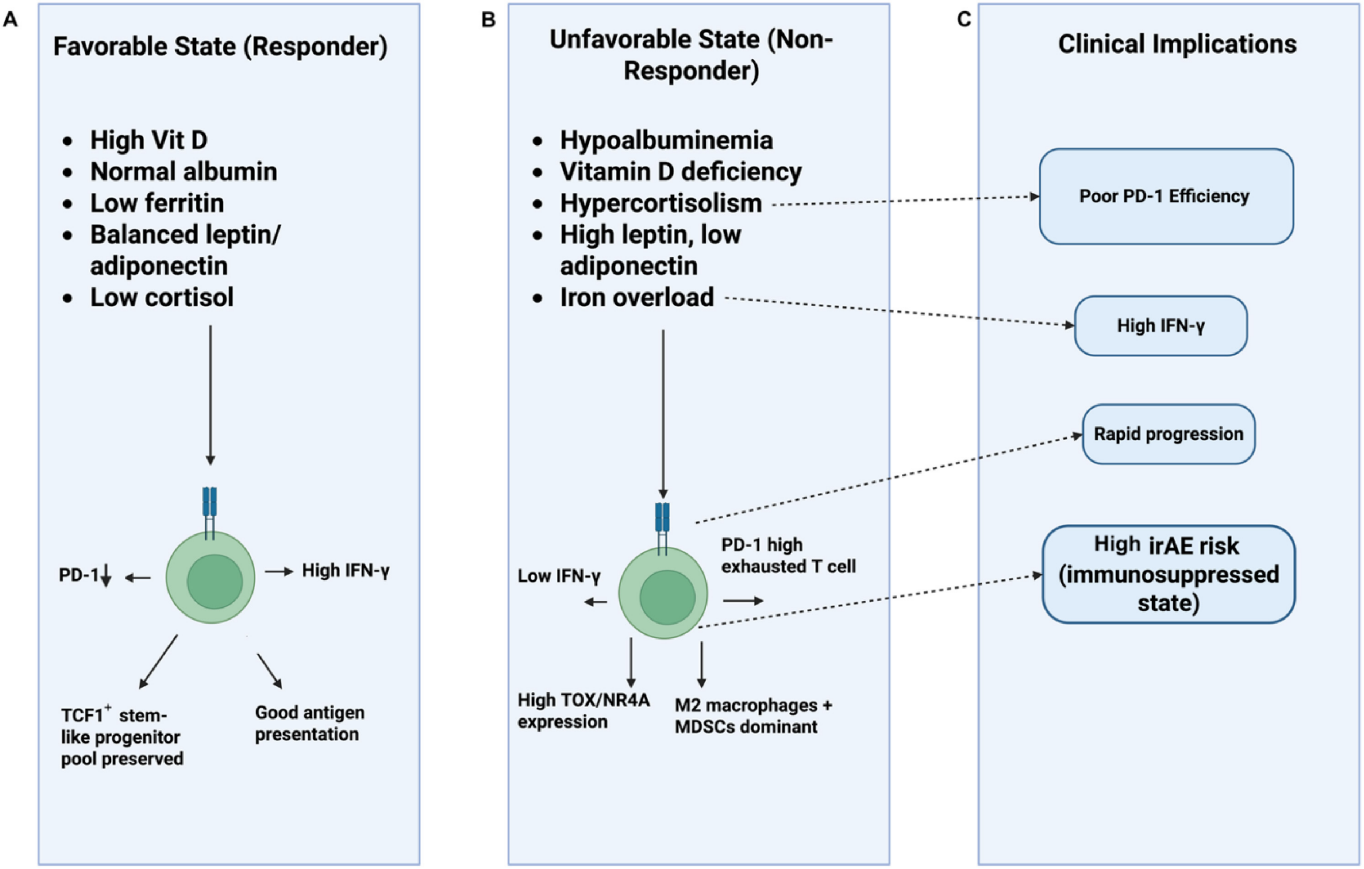
Endocrine-nutritional modulation of T-cell fate and immune checkpoint therapy response. This schematic illustrates how metabolic, endocrine, and nutritional states shape T-cell functionality and influence clinical outcomes during PD-1/PD-L1 checkpoint inhibition. **(A)** Track A (Favorable State) shows conditions associated with better therapeutic response including sufficient vitamin D, normal albumin, balanced adipokines, low ferritin, and reduced cortisol promoting *PD-1^low^* effector activity, preserved *TCF1*^+^ progenitor pools, robust IFN-γ production, and efficient antigen presentation. **(B)** Track B (Unfavorable State) depicts deficiencies and dysregulations such as hypoalbuminemia, vitamin D deficiency, hypercortisolism, adipokine imbalance, and iron overload, which drive *PD-1^high^* T-cell exhaustion, elevated TOX/NR4A expression, reduced IFN-γ, and a suppressive myeloid milieu dominated by M2 macrophages and MDSCs. **(C)** Track C (Clinical Implications) summarizes downstream consequences, linking these states to poor PD-1 inhibitor efficacy, rapid disease progression, and higher incidence of immune-related adverse events due to overall immunosuppression.

Vitamin D/VDR signaling inhibits exhaustion genes (PDCD1, HAVCR2) via direct binding to super-enhancers and promotes stem-like TCF1 + PD-1int progenitors capable of reinvigoration upon PD-1 blockade (12, 124). Zinc deficiency impairs ZAP-70/LCK signaling and IL-2 production, accelerating exhaustion; zinc supplementation restores cytotoxicity and reduces PD-1 expression (187). Arginine and tryptophan availability are critical: tumor/MDSC-derived ARG1/IDO1 deplete these amino acids, activating GCN2/ATF4 stress pathways that upregulate PD-1 and inhibit memory formation; arginine supplementation or IDO inhibitors prevent exhaustion and enhance PD-1 blockade (95, 96). Figure 5 summarizes how endocrine and nutritional factors shape T-cell states and influence PD-1/PD-L1 therapy outcomes.

### 7.2 Effects on antigen-presenting cells and myeloid compartment

Endocrine–nutritional cues reprogram APCs and MDSCs, determining co-stimulatory capacity and immunosuppressive polarization. Vitamin D is a master regulator of DC maturation: 1,25(OH)2D induces tolerogenic phenotype (↑CD14, ↓CD80/86, ↑IL-10) in steady state but enhances cross-presentation and CXCL10 production in inflammatory contexts, promoting CD8^+^ priming and ICI efficacy (188, 189). Zinc and selenium support lysosomal function and ROS detoxification in DCs, preserving MHC-II expression and co-stimulatory molecule expression; deficiency impairs antigen presentation and favors MDSC expansion (94).

Cortisol and leptin drive M2/macrophage polarization via PPARγ/STAT6 and suppress MHC-II/CD86 on APCs, limiting T-cell priming (65). In obesity, leptin-rich marrow adipocytes secrete IL-6/TGF-β that expand PMN-MDSCs expressing PD-L1 and ARG1, directly inhibiting CD8^+^ T-cell function (19). Iron dysregulation (high ferritin/hepcidin) promotes MDSC accumulation and M2 polarization while starving T cells; ferroptosis induction in MDSCs enhances ICI responses (179). These myeloid shifts create a “cold” pre-treatment state resistant to PD-1/PD-L1 blockade.

### 7.3 Modulation of tumor microenvironment in hematologic cancers

The bone marrow TME in lymphomas and myeloma is uniquely sensitive to endocrine–nutritional inputs due to high adipocyte content, chronic IL-6 signaling, and nutrient competition for glucose/amino acids (190). Marrow adipose tissue (MAT) expands dramatically in MM and aggressive lymphomas, secreting leptin, adiponectin (often reduced), and free fatty acids that reprogram resident immune cells (191). Leptin from MAT drives PD-L1 expression on myeloma cells via JAK2/STAT3 and promotes MDSC/Treg infiltration, while adiponectin deficiency removes CD8^+^-supportive signals (192).

Vitamin D deficiency, prevalent in MM due to bone disease, exacerbates marrow immunosuppression by reducing CXCL10-mediated T-cell trafficking and enhancing RANKL-driven osteoclastogenesis that releases TGF-β (193). Insulin/IGF-1 hyperactivation in obese myeloma patients upregulates PD-L1 on plasma cells and fosters “exhausted-like” marrow-resident T cells with high TOX expression (194). Zinc/selenium depletion—common from poor intake/malabsorption—impairs NK-cell degranulation against RS cells in cHL, contributing to the immunosuppressive niche (93). Thus, unfavorable endocrine–nutritional profiles reinforce marrow as an immune-privileged sanctuary, explaining limited PD-1 monotherapy success in MM vs. cHL (where 9p24.1 amplification overrides some constraints).

### 7.4 Interplay with cytokine and chemokine networks

Endocrine–nutritional status orchestrates cytokine networks that determine whether PD-1 blockade triggers productive IFN-γ-driven tumor control or futile inflammation. IFN-γ induces PD-L1 on tumor/APCs to enable adaptive resistance; however, chronic IL-6 (driven by obesity, stress, malnutrition) trans-signals STAT3 to upregulate alternative checkpoints (TIM-3, LAG-3) and anti-apoptotic genes, blunting IFN-γ efficacy (97). Cortisol suppresses IFN-γ/IL-2 while elevating IL-10/TGF-β, shifting from Th1 to Treg-polarizing milieu (105).

Vitamin D and short-chain fatty acids (from fiber-rich diets enhance IFN-γ/CXCL9/10/11 production by DCs, promoting CD8^+^ T-cell trafficking and PD-1 blockade sensitivity (100). Leptin amplifies IL-6/TNF-α loops that sustain exhaustion, whereas adiponectin dampens them (83). Arginine depletion inhibits iNOS-derived NO needed for chemokine receptor expression, impairing T-cell migration; restoration reinvigorates cytokine networks (95).

### 7.5 Influence on immune-related adverse events

Paradoxically, endocrine–nutritional factors that impair antitumor efficacy often protect against severe irAEs by limiting systemic immune activation. Thyroid irAEs (most common endocrine toxicity, 10–40%) reflect breakthrough autoimmunity: patients developing thyroiditis exhibit higher baseline T-cell reactivity and IFN-γ signatures, translating to both superior tumor control and autoimmunity risk (69, 195). Vitamin D sufficiency correlates with increased irAE incidence (particularly colitis, pneumonitis) but improved survival, likely via enhanced T-cell reinvigoration (125).

Obesity/leptin excess is associated with higher rates of severe irAEs, possibly via tonic mTOR activation, lowering activation threshold (196). Conversely, malnutrition (low albumin, PNI) predicts fewer irAEs but worse oncologic outcomes, reflecting globally suppressed immunity (197). Baseline GC use or endogenous hypercortisolism dramatically reduces irAE risk but abolishes ICI benefit (106). Thus, the same mechanisms driving resistance (exhaustion promotion) often confer irAE protection, while “fit” profiles yield both better tumor responses and manageable toxicity.

## 8 Clinical applications and future directions

Pre-treatment endocrine–nutritional optimization represents a low-cost, low-toxicity strategy to enhance PD-1/PD-L1 blockade efficacy, with emerging prospective data supporting vitamin D repletion, metformin for insulin resistance, and structured nutritional support. Although most evidence derives from solid tumors, mechanistic overlap and real-world hematologic cohorts suggest translatability, particularly in cachexia-prone lymphomas and myeloma. Integration of simple blood-based composites (e.g., PNI + vitamin D + ferritin) with tumor biomarkers could enable precision supportive care, converting “unfit” hosts into better responders.

### 8.1 Developing clinically feasible endocrine–nutritional assessments

Routine, low-cost blood tests (albumin, prealbumin, CRP, vitamin D, ferritin, zinc, selenium, morning cortisol, TSH/free T4, fasting glucose/insulin or C-peptide, leptin/adiponectin ratio) combined with CT-based body composition (skeletal muscle index, subcutaneous/visceral fat) provide a feasible “immuno-nutritional panel” implementable in standard oncology practice (183, 198). PNI, CONUT score, and GPS require only CBC and chemistry, achieving high stratification power in ICI cohorts (136). Point-of-care or multiplex assays for adipokines and micronutrients are emerging, with turnaround < 48 h. In hematologic settings, adding serum-free light chains or hepcidin may refine marrow-specific risk (199).

### 8.2 Can we intervene before therapy?

Prospective trials increasingly support pre-emptive correction of deficiencies to augment ICI outcomes.

#### 8.2.1 Vitamin D correction

Vitamin D deficiency (<20 ng/mL) is correctable with 50,000 IU weekly loading followed by 2,000–4,000 IU daily maintenance. A 2023 prospective cohort (*n* = 200 + ICI patients) showed systematic supplementation improved ORR from 36 to 56% and median PFS from 5.8 to 11.3 months, with greater CD8^+^ T-cell reinvigoration and reduced severe irAEs (125). A multicenter analysis confirmed that higher serum 25(OH)D and supplementation independently predicted superior survival in PD-1-treated solid tumors, with similar trends in lymphoma subsets (87).

#### 8.2.2 Iron balance

High ferritin and functional iron deficiency predict resistance via MDSC expansion. Careful IV iron in true deficiency or hepcidin inhibitors (preclinical) may help, but excess iron worsens outcomes; guidelines recommend avoiding overload and monitoring transferrin saturation (200, 201).

#### 8.2.3 Nutrition support

High-protein oral supplementation + resistance exercise reverses sarcopenia and improves PNI in 4–8 weeks. Mediterranean or high-fiber diets enrich responder microbiomes (Akkermansia, Faecalibacterium), with a 2025 phase II trial showing high-fiber intervention increased ORR by 20–30% in ICI patients (100, 202). Enteral/parenteral nutrition in severe malnutrition stabilizes weight and albumin pre-ICI (203).

#### 8.2.4 Hormonal optimization

Metformin in insulin-resistant patients activates AMPK, reduces MDSCs, and potentiates PD-1 blockade; phase II trials in NSCLC/melanoma report ORR ↑20–40% and PFS HR 0.6–0.7 when added to nivolumab/pembrolizumab (59, 204, 205). Thyroid replacement in hypothyroidism and physiologic hydrocortisone in adrenal insufficiency are standard; androgen deprivation in males is under investigation (75).

### 8.3 Precision supportive care to augment immunotherapy

Risk-stratified supportive algorithms, intensive intervention for high-risk profiles (low PNI + vitamin D deficiency + sarcopenia) could increase population-level ICI benefit by 15–30% at minimal cost (13). Ongoing trials combine metformin + high-protein diet + vitamin D in “unfit” patients starting PD-1 therapy.

### 8.4 Integration with genomic/immune biomarkers

Composite scores merging host (PNI/GPS + vitamin D) with tumor factors (PD-L1 TPS, TMB, 9p24.1 status in cHL) outperform either alone, achieving > 85% accuracy in predicting durable response in retrospective validation (206, 207). Machine learning models incorporating sarcopenia, leptin, and IFN-γ signature are in development for hematologic ICI combinations (208).

### 8.5 Proposed algorithm for clinical translation

1. Baseline assessment (day 14 to day 0): blood panel + CT body composition.
2. Risk stratification: low-risk (PNI > 45, vit D > 30, no sarcopenia) → proceed to ICI; high-risk → intervene 4–8 weeks (vitamin D repletion, metformin if HOMA-IR > 2.5, high-protein ONS + exercise, consider short-course nutrition if CONUT ≥ 5.
3. Re-assess panel pre-cycle 1; initiate ICI ± continued support.
4. Monitor response and irAEs; escalate nutrition if progression/cachexia (209).

**FIGURE 6:**
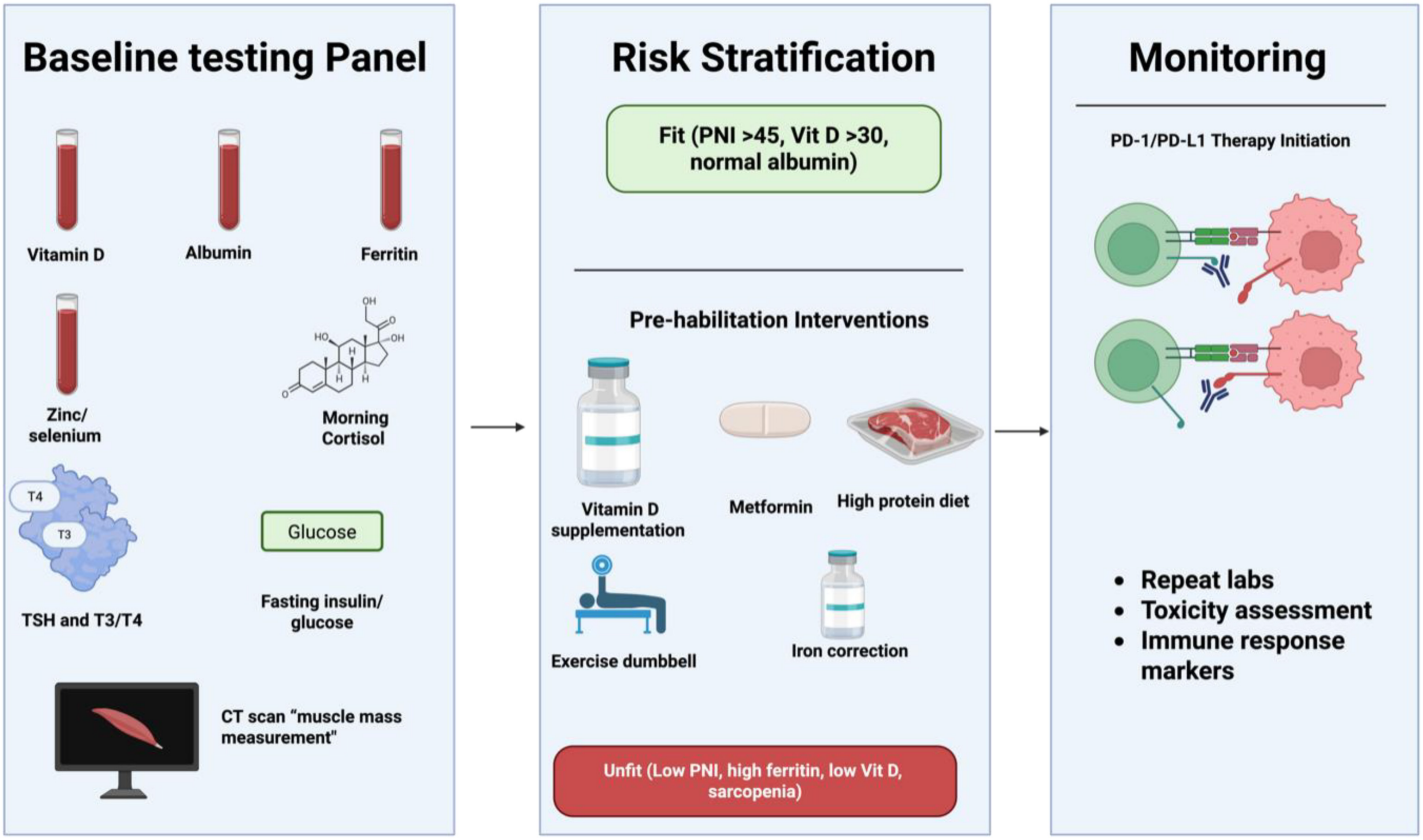
Clinical workflow for endocrine–nutritional optimization before PD-1/PD-L1 immunotherapy. This algorithm summarizes a structured pre-treatment approach designed to enhance immune checkpoint inhibitor efficacy. Step 1 (Baseline Testing Panel): Comprehensive assessment including serum vitamin D, albumin, ferritin, zinc/selenium, morning cortisol, thyroid function (TSH, free T4), fasting insulin–glucose, and imaging-derived muscle mass evaluation. Step 2 (Risk Stratification): Patients are categorized as *Fit* (e.g., PNI > 45, vitamin D > 30 ng/mL, normal albumin) or *Unfit* based on markers of malnutrition, inflammation, micronutrient deficiencies, and sarcopenia. Step 3 (Pre-habilitation Interventions): Targeted corrective strategies—vitamin D repletion, metformin use for metabolic optimization, high-protein dietary support, structured exercise, and cautious management of iron overload or deficiency. Step 4 (Therapy Initiation): Commencement of PD-1/PD-L1 blockade with antibody–receptor interaction illustrated. Step 5 (Monitoring): Ongoing evaluation through repeat laboratory testing, toxicity screening, and immunologic response tracking to ensure optimal therapeutic benefit.

This pragmatic framework, adaptable to resource settings, warrants prospective validation in phase III adjuvant/neo-adjuvant ICI trials in lymphoma and myeloma. Figure 6 outlines the proposed clinical workflow for endocrine–nutritional optimization before initiating PD-1/PD-L1 therapy. To operationalize the proposed assessment model, we provide a structured framework summarizing endocrine and nutritional biomarkers that can be incorporated into standard pre-treatment evaluation for ICI candidates (Table 6). These markers are chosen based on availability, clinical relevance, and potential to inform risk stratification and supportive interventions.

**TABLE 6:** Pre-treatment endocrine and nutritional assessment framework for ICI candidates.

Assessment category	Specific markers/indices	Method/measurement	Clinical interpretation and actions
Baseline nutritional status	Serum albumin	Routine CMP	↓ Albumin → risk of poor outcomes; consider dietetics referral and nutritional support
	Prealbumin	Routine lab panel	Rapid indicator of protein status; monitor trends
	BMI/weight change	Clinical measurement	Significant weight loss → malnutrition; tailored intervention
Inflammation/immune Status	Neutrophil–lymphocyte ratio (NLR)	CBC differential	↑ NLR → systemic inflammation; enhanced surveillance
	Prognostic Nutritional Index (PNI)	Albumin + lymphocyte count	Low PNI → higher risk; supportive care recommended
	C-reactive protein (CRP)	Serum assay	↑ CRP → inflammation; monitor and interpret in context
Endocrine parameters	Fasting glucose/insulin	Serum assays	Hyperglycemia/insulin resistance → metabolic risk; optimize glycemic control
	Thyroid function (TSH, free T4)	Serum assays	Hypo/hyperthyroidism → endocrine correction before ICI
	Cortisol (morning)	Serum assay	Abnormal → evaluate HPA axis; adjust therapy accordingly
	Vitamin D	Serum 25-OH vit D	Deficiency → replete; supportive immune function
Composite scoring	Glasgow Prognostic Score (GPS)	CRP + albumin	Risk stratification and prognostic grouping
	CONUT score	Albumin + lymphocytes + cholesterol	Low score → favorable prognosis; guide nutrition focus
Dynamic monitoring	Repeat key markers at 6–8 weeks	CBC, CMP, CRP, albumin	Changes may signal response or toxicity; adapt care plan

Table 6 presents a structured pre-treatment endocrine and nutritional assessment framework designed to optimize patient selection and risk stratification prior to initiation of ICI therapy. This framework integrates routinely accessible hormonal, metabolic, and nutritional parameters to capture baseline host immunometabolic competence, which is increasingly recognized as a determinant of ICI responsiveness and toxicity. Pre-existing endocrine abnormalities such as subclinical thyroid dysfunction, adrenal axis dysregulation, glucose intolerance, and sex hormone imbalance—may predispose patients to altered immune activation thresholds, exaggerated immune-related adverse events, or premature immune exhaustion, thereby influencing both therapeutic efficacy and tolerability. Concurrently, nutritional and body composition metrics including BMI, sarcopenia indices, serum albumin, prealbumin, PNI, and inflammation-linked nutritional scores serve as surrogates for systemic energy reserve, muscle-derived immunomodulatory signaling, and chronic inflammatory burden. By synthesizing these dimensions, the framework outlined in Table 6 moves beyond single biomarker evaluation toward a holistic, host-centered stratification model, enabling identification of patients who may benefit from prehabilitation strategies such as nutritional supplementation, endocrine correction, or metabolic optimization before ICI exposure. Importantly, this approach supports a shift from reactive management of immune-related complications to proactive personalization of immunotherapy, positioning endocrine–nutritional profiling as a pragmatic and scalable tool for improving clinical outcomes.

### 8.6 Clinical feasibility and implementation considerations

Although endocrine–nutritional signatures show strong biological plausibility and prognostic relevance, their clinical utility depends on feasibility, standardization, and cost-effectiveness. Importantly, many of the proposed biomarkers are already routinely measured in standard oncology practice, facilitating near-term translation without the need for specialized assays (210, 211).

#### 8.6.1 Biomarker availability and cost

Core nutritional and inflammatory markers such as serum albumin, CRP, complete blood count, ferritin, and glucose are inexpensive, widely available, and standardized across laboratories. Composite indices derived from these parameters, including the PNI, GPS, and CONUT score, require no additional testing and can be calculated retrospectively or prospectively at minimal cost. Endocrine parameters such as TSH, free T4, fasting glucose, and insulin are similarly routine and inexpensive, while vitamin D testing is increasingly standardized and cost-effective in many healthcare systems (212). In contrast, certain biomarkers such as leptin/adiponectin ratios, selenium levels, or morning cortisol may be less routinely available and incur higher costs or longer turnaround times. These markers may therefore be best reserved for research settings or high-risk patients, rather than universal screening, until stronger prospective validation is achieved.

#### 8.6.2 Inter-laboratory variability and standardization

Variability in assay platforms and reference ranges represents a key challenge, particularly for biomarkers such as vitamin D, ferritin, cortisol, and adipokines. Circadian variation (e.g., cortisol), acute inflammatory states, and concurrent medications (e.g., corticosteroids, thyroid hormone replacement) can further confound interpretation. To mitigate these issues, standardized timing (e.g., morning sampling for cortisol), repeated baseline measurements, and use of clinically established cut-offs rather than institution-specific percentiles are recommended.

Importantly, composite indices that integrate multiple parameters (e.g., PNI or GPS) are inherently more robust to single-measurement variability and may offer superior reproducibility across institutions compared with single biomarkers.

#### 8.6.3 Clinical workflow integration

From a practical standpoint, endocrine–nutritional assessment can be incorporated into routine pre-immunotherapy evaluation without delaying treatment initiation. Most markers can be obtained within standard pre-treatment laboratory panels, allowing risk stratification within days. Patients identified as “high-risk” based on these profiles may benefit from early supportive interventions (nutritional optimization, vitamin D repletion, metabolic control) without altering oncologic treatment selection (213–215).

#### 8.6.4 Regulatory and translational considerations

Unlike tumor genomic biomarkers, endocrine–nutritional markers do not require regulatory approval as companion diagnostics, lowering barriers to implementation. However, prospective validation studies and harmonized reporting standards are needed before these signatures can be adopted as decision-modifying tools. Future clinical trials incorporating immune checkpoint inhibitors in hematologic malignancies should prospectively collect these parameters to define optimal cut-offs, timing, and intervention strategies. Overall, the low cost, wide availability, and biological relevance of endocrine–nutritional biomarkers make them attractive candidates for real-world clinical application, particularly as adjunctive tools to complement tumor-intrinsic predictors rather than replace them.

### 8.7 Prospective cohort design, integrative biomarker panels, and AI-based prediction models

Advancing endocrine–nutritional signatures for predicting PD-1/PD-L1 blockade efficacy in hematologic malignancies requires robust prospective cohort designs to overcome retrospective limitations, such as selection bias and incomplete data. Prospective studies enable standardized baseline assessments of hormones (e.g., cortisol, thyroid), nutrients (e.g., vitamin D, zinc), and composites like the PNI, alongside longitudinal monitoring of ORR, PFS, and irAEs. The PROVIDENCE study (2023), a prospective observational trial in advanced cancer patients on ICIs, demonstrated that systematic vitamin D supplementation improved ORR from 36 to 56% and median PFS from 5.8 to 11.3 months, while reducing thyroid irAEs. In hematologic contexts, adapting this approach could involve cohorts of relapsed/refractory cHL or MM patients, stratifying by marrow infiltration and prior therapies, which often induce cachexia and dysregulate the immuno-endocrine axis. Challenges include heterogeneity in disease subtypes and high attrition, but powering for endpoints like 12-month PFS could yield level I evidence, informing guidelines for pre-treatment interventions (125, 216–218).

Integrative biomarker panels enhance predictive accuracy by combining endocrine (e.g., insulin/IGF-1), nutritional (e.g., albumin, ferritin), inflammatory (e.g., CRP/neutrophil-lymphocyte ratio), and body composition metrics (e.g., sarcopenia via CT). Panels like the Systemic Immune-Inflammation Index predict poorer OS/PFS in ICI-treated cancers, reflecting T-cell exhaustion and myeloid suppression. In hematologic malignancies, the Systemic Immunologic Readiness Score integrating GPS, PNI, and microbiome data stratifies “fit” vs. “unfit” patients, with high scores correlating to superior ORR in cHL. Preclinical models validate synergy: hypercortisolism plus hypoalbuminemia triples resistance via mTOR/IFN-γ inhibition. Hematology-tailored panels could incorporate hepcidin for iron dysregulation in MM, enabling targeted therapies like metformin or nutritional support to boost efficacy (219–223). AI-based prediction models revolutionize personalization by integrating multi-omics (genomics, metabolomics) with endocrine–nutritional data. Machine learning algorithms, such as random forests, predict ICI response with > 85% accuracy using routine blood parameters in pan-cancer cohorts. In 2025 updates, AI models for PD-1/PD-L1 efficacy in hematologic malignancies incorporate PD-L1 expression estimation from H&E slides, forecasting PFS in NSCLC analogs adaptable to lymphomas. Federated learning across registries (e.g., LYSA) handles real-time inputs like cortisol rhythms, identifying non-responders for adjunctives. Ethical issues like bias require validation in trials (e.g., NCT05352750), but these models could optimize r/r settings, addressing gaps in tumor-centric biomarkers (224–228).

### 8.8 Future directions and global applicability

Future studies should address ethnic and geographic variability in pre-treatment endocrine–nutritional signatures to enhance the global applicability of these predictive biomarkers for PD-1/PD-L1 blockade. Baseline levels of key components such as vitamin D, iron stores, thyroid hormones, cortisol, and nutritional indices vary substantially across populations due to differences in latitude, sunlight exposure, dietary patterns, genetic polymorphisms, socioeconomic factors, and healthcare access. For example, vitamin D deficiency is highly prevalent in populations residing at higher latitudes and among individuals with increased skin pigmentation, cultural sun-avoidance practices, or limited dietary fortification, potentially influencing immune competence and immunotherapy responsiveness. Similarly, regional differences in iron deficiency or overload, micronutrient availability (e.g., zinc and selenium), and sarcopenia prevalence may differentially shape systemic immunologic readiness across ethnic groups. Prospective, multi-ethnic cohorts are therefore needed to define population-specific reference ranges and thresholds for these signatures, as well as to determine whether their predictive value is consistent across diverse hematologic malignancies and treatment settings. Incorporating geographic, lifestyle, and genetic modifiers into predictive models may enable more accurate patient stratification and support the development of tailored nutritional or endocrine interventions to optimize immunotherapy outcomes worldwide.

## 9 Challenges

Translating endocrine–nutritional signatures into reliable predictors of PD-1/PD-L1 blockade outcomes faces substantial hurdles that currently limit clinical adoption (229). Disease heterogeneity poses a primary challenge: hematologic malignancies exhibit profound inter- and intra-patient variability in metabolic phenotypes, e.g., Reed-Sternberg cells in classical Hodgkin lymphoma drive unique leptin-rich microenvironments, while MM features IGF-1-dominated marrow niches, making universal cut-offs elusive and requiring disease-specific validation (190). Confounders further complicate interpretation: concomitant corticosteroids (used in 20–40% of patients for symptom palliation or irAE management) potently suppress T-cell function and confound inflammatory/nutritional indices, with baseline use independently worsening PFS/OS (HR 1.5–2.5) yet often unavoidable in lymphoproliferative disorders (106, 108). Cancer cachexia, affecting 50–80% of advanced patients, drives hypoalbuminemia and sarcopenia through IL-6-mediated hypercatabolism rather than pure malnutrition, masking true immunologic readiness (168). Comorbidities (diabetes, obesity, autoimmune disease) introduce bidirectional bias, as insulin resistance promotes exhaustion while obesity paradoxically protects via leptin-sensitive subsets (81, 230).

### 9.1 Knowledge gaps

Endocrine markers display marked circadian variability, with cortisol peaks at 06:00–08:00 and nadirs at midnight, with flattened rhythms prognostic of poorer survival in multiple cancers necessitating standardized morning sampling that is rarely enforced in retrospective cohorts (231, 232). The evidence base remains predominantly observational, with few prospective interventional trials: vitamin D repletion studies show promise but are limited by small size and lack of randomization, while nutritional prehabilitation lacks phase III data in ICI settings (233, 234). Finally, the absence of consensus cut-offs hampers comparability. Albumin thresholds range 30–35 g/L, PNI 40–50, vitamin D 20–30 ng/mL across studies reflecting population-specific inflammation burdens and assay differences (235). These gaps underscore the need for large, prospective, biomarker-driven trials incorporating serial sampling, confounder adjustment, and harmonized thresholds to establish causative links and enable routine clinical use (236, 237).

## 10 Limitations

While this review synthesizes current evidence on endocrine–nutritional signatures and their relevance in hematologic malignancies, several limitations should be acknowledged.

### 10.1 Heterogeneity of evidence

The mechanistic frameworks we describe draw from studies with diverse designs, patient populations, and endpoints. Biomarker associations identified in retrospective cohorts may not fully capture causal relationships and may reflect underlying confounders. Moreover, different studies employ varying assay platforms, cut-offs, and composite index definitions, which complicates direct comparisons and quantitative synthesis.

### 10.2 Small and selective cohorts

Several mechanistic insights and prognostic associations derive from small or single-institution cohorts. These studies may be underpowered to detect modest effects, and findings may not generalize across broader demographic or disease subgroups. The limited sample sizes also restrict our ability to evaluate interactions among biomarkers, treatment regimens, and clinical outcomes.

### 10.3 Speculative biological pathways

Although many endocrine–nutritional pathways have plausible roles in modulating immune function and treatment response, some proposed mechanisms remain speculative due to incomplete experimental validation. For example, the interplay between adipokines, systemic metabolism, and antitumor immunity has strong preclinical support but requires further confirmation in longitudinal clinical studies with standardized sampling.

### 10.4 Measurement variability and timing

As discussed above, biomarker measurements may vary with circadian rhythms, acute illness, and assay methodology. Most published studies do not report standardized timing or repeated measures, which could influence observed associations. This limitation is particularly relevant for hormones with diurnal variation or for markers influenced by concurrent medications (e.g., corticosteroids).

The manuscript’s reliance on retrospective cohorts introduces significant biases, including selection bias from non-randomized patient inclusion, confounding by unmeasured variables (e.g., comorbidities or prior therapies), and incomplete data on endocrine-nutritional profiles, potentially overestimating associations with PD-1/PD-L1 outcomes. For instance, real-world studies in hematologic malignancies often lack standardized biomarker assessments, leading to inconsistent findings on predictors like hypoalbuminemia or vitamin D deficiency. Small sample sizes further compound issues, limiting statistical power and increasing the risk of spurious results or type II errors, as seen in early-phase ICI trials where cohorts under 100 patients fail to detect subgroup effects in diverse hematologic subtypes. Overrepresentation of Asian populations in cited studies (e.g., higher EGFR mutation rates influencing immunotherapy responses) may reduce generalizability to Western cohorts, where genetic and environmental factors differ, potentially skewing efficacy estimates for endocrine signatures (238–242). Future research should prioritize prospective, multicenter trials with larger, diverse cohorts to mitigate biases and validate biomarkers like PNI or GPS. Strategies include integrating multi-omics (e.g., metabolomics with nutritional data), machine learning for predictive models, and global collaborations to ensure ethnic representation, enhancing translational applicability in hematologic settings (226, 243–245).

### 10.5 Future directions to mitigate limitations

Prospective, multicenter studies with standardized protocols are needed to validate promising signatures and to clarify causal mechanisms. Harmonized reporting standards and collaborative consortia will facilitate meta-analyses that overcome individual cohort limitations. Integration of mechanistic studies with clinical outcomes will strengthen the biological rationale and translational potential of endocrine–nutritional markers. By acknowledging these constraints, our review aims to present a balanced interpretation of current evidence while highlighting opportunities for future research.

## 11 Conclusion

In summary, pre-treatment endocrine–nutritional signatures emerge as pivotal, modifiable predictors of PD-1/PD-L1 blockade efficacy in hematologic malignancies, extending beyond tumor-centric biomarkers. Dysregulations in cortisol, thyroid hormones, sex steroids, insulin/IGF-1, adipokines, vitamin D, zinc, and protein status converge to impair T-cell reinvigoration, antigen presentation, and marrow microenvironment permissiveness, driving heterogeneous responses. Integrated multi-marker panels, such as GPS or PNI combined with vitamin D and sarcopenia indices, provide robust prognostic stratification, with potential to guide personalized interventions like hormone modulation, micronutrient repletion, metformin, or dietary optimization. While evidence is strongest in cHL and PMBCL, applicability to refractory MM and leukemias warrants prospective trials to validate signatures, standardize cut-offs, and test adjunctive strategies. By addressing these host factors, we can convert immunologically “unfit” patients to responders, enhancing durable remissions and reducing resistance in this challenging landscape. Future research should incorporate machine learning for dynamic profiling and microbiome integration to fully harness endocrine–nutritional crosstalk for immunotherapy success.
